# Toward a ToxAtlas of Carbon-Based Nanomaterials: Single-Cell RNA Sequencing Reveals Initiating Cell Circuits in Pulmonary Inflammation

**DOI:** 10.1021/acsnano.5c12054

**Published:** 2025-11-03

**Authors:** Carola Voss, Lianyong Han, Meshal Ansari, Maximilian Strunz, Verena Haefner, Ilias Angelidis, Christoph H. Mayr, Trine Berthing, Qiaoxia Zhou, Eva M. Guenther, Osama Huzain, Otmar Schmid, Ulla Vogel, Janine Gote-Schniering, Svenja Gaedcke, Fabian J. Theis, Herbert B. Schiller, Tobias Stoeger

**Affiliations:** † Institute of Lung Health and Immunity (LHI), Comprehensive Pneumology Center (CPC), Helmholtz Center Munich, Neuherberg 85764, Germany; ‡ 9177 German Center for Lung Research (DZL), Munich 81377, Germany; § Hannover Medical School, Clinic for Cardiac, Thoracic, Transplantation and Vascular Surgery, Leibniz Research Laboratories for Biotechnology and Artificial Organs (LEBAO), Hannover 30625, Germany; ∥ Biomedical Research in Endstage and Obstructive Lung Disease Hannover (BREATH), German Center for Lung Research (DZL), Hannover 30625, Germany; ⊥ Department of Computational Health, Institute of Computational Biology, Helmholtz Center Munich, Neuherberg 85764, Germany; # 2686 National Research Centre for the Working Environment, Copenhagen 2100, Denmark; ∇ Institute of Experimental Pneumology, LMU University Hospital, Ludwig-Maximilians University, Munich 81377, Germany; ○ Department of Rheumatology and Immunology, Department of Pulmonary Medicine, Allergology and Clinical Immunology, Inselspital, Bern University Hospital, University of Bern, Bern 3012, Switzerland; ◆ Lung Precision Medicine, Department for BioMedical Research, University of Bern, Bern 3012, Switzerland; ¶ Department of Respiratory Medicine and Infectious Diseases, Hannover Medical School, Hannover 30625, Germany; †† School of Life Sciences Weihenstephan, Technical University of Munich, Munich 35354, Germany; ‡‡ School of Computing, Information and Technology, Technical University of Munich, Munich 35354, Germany; §§ Wellcome Sanger Institute, Wellcome Genome Campus, Cambridge CB10 1SA, U.K.; ∥∥ Research Unit for Precision Regenerative Medicine, Helmholtz Center Munich, Munich 81377, Germany; ⊥⊥ Comprehensive Pneumology Center Munich (CPC), German Center for Lung Research (DZL), Munich 81377, Germany

**Keywords:** scRNA-seq, pulmonary inflammation, nanomaterials, mode of action (MoA)/cell circuits, ToxAtlas, predictive assays, respiratory toxicology

## Abstract

Understanding how nanomaterial properties drive acute lung inflammation is critical for the development of safer materials, but for low solubility carbon-based nanomaterials (CBNs) the initiation of the inflammatory response is still poorly understood. Leveraging single-cell RNA sequencing of mouse lungs, 12 h after intratracheally instillation with different CBN spherical carbon nanoparticles (CNP), tangled double-walled (DWCNT), and rigid multiwalled carbon nanotubes (MWCNT) and lipopolysaccharide (LPS) as positive control, we identified 41 cell states and delineated material-specific molecular initiation events at single-cell resolution. CBN doses were chosen to cause equal levels of moderate inflammation, assessed by airspace neutrophilia, and exposure-triggered cellular activation was tested for *in vitro* reproducibility. To advance future development of cell-based assays, we developed a webtool, ToxAtlas, mapping CBN-specific gene responses of interest. Despite chemical similarity, CBN elicited distinct inflammatory cytokine and cell responses via different modes of action. CNP triggered neutrophilia through alveolar epithelial activation and *Cxcl1* and *Csf2* expression but without apparent cell damage or macrophage activation. In contrast, CNT induced epithelial and macrophage damage, with alarmin release (IL-1α, IL-33) dominating the MWCNT response. DWCNT caused alveolar epithelial injury, and pro-inflammatory macrophage and fibroblast-derived monocyte attractant (*Ccl2*, *Ccl7*) activation. Our initiating cell circuits identify epithelial as well as early fibroblast activation, especially from alveolar type 2 cell-adjacent lipofibroblasts, as central to orchestrating the initiation of CBN-induced inflammation. These findings support the role of mesenchymal cells in early pulmonary defense, eventually priming chronic inflammation, a known cause of MWCNT exposure.

## Graphical abstract

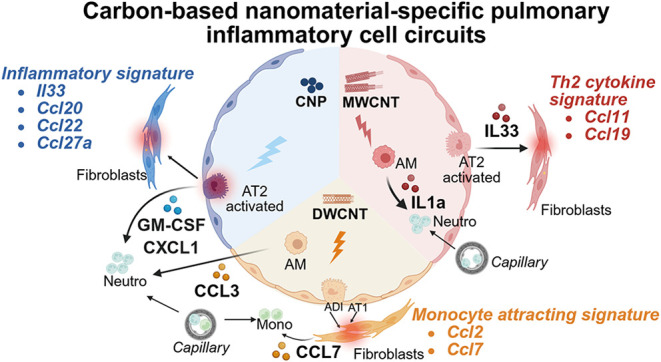

The ever-growing production and application of nanomaterials has raised increasing concern about their potential effects on human health, particularly via inhalation exposure. The respiratory tract represents one of the most vulnerable entry routes for particulate matter, and history has provided striking examples of inhaled fibrous or nanoscale materials causing severe pulmonary disease. Asbestos exposure has been directly linked to asbestosis, mesothelioma, and lung cancer, pointing at the susceptibility of the respiratory barrier to fiber-like particles.^1,2^ Similarly, ultrafine particles derived from air pollution can penetrate deeply into the fragile alveolar region of the lungs, where they are associated with detrimental outcomes including lung cancer.^3–5^ A key factor for the biological impact of nanomaterials is their high surface area-to-mass ratio, which promotes extensive interactions with biological systems and excessive generation of reactive oxygen species.^6^ In addition, physicochemical properties such as particle size, shape, and biopersistence critically determine toxicological effects.^7^ Acute and transient pulmonary inflammation is the most common dose-dependent response that can be initiated *in vivo* by almost any nanomaterial. We have previously shown that, for poorly soluble, low-toxicity particles, the deposited particle surface area in the lung is the most relevant determinant of acute pulmonary inflammation.^8,9^ Additionally, continuous exposure to biopersistent carbon-based nanomaterials (CBN) can result in chronic lung injury, tissue remodeling, and progression toward pulmonary fibrosis or cancer, as exemplified by asbestos and rigid, needle-shaped carbon nanotubes (CNT).^7,10^ In the absence of sufficiently predictive *in vitro* assays, hazard and risk assessment of inhaled CBN still relies heavily on animal studies. *In vivo* inhalation or instillation enables evaluation of global pulmonary toxicity and has been critical for identifying adverse outcome pathways (AOPs) relevant to respiratory health.^11^ The AOP concept links molecular initiating events to adverse health outcomes and is central to the design of new animal-free safety assessment strategies.

Recently, transcriptomics has become an indispensable tool in toxicology to dissect global gene expression changes and pathway activation. In this context, several studies using bulk transcriptomics approaches have provided critical insights into pulmonary responses to CNT. For example, Bornholdt et al. performed genome-wide mapping of transcriptional start sites and enhancer regions in mouse lungs 24 h after high-dose MWCNT instillation, revealing robust induction of key inflammatory genes and identifying loci associated with enhancer activation. This resource has served as a valuable reference for biomarkers of CNT-induced inflammation and for designing relevant *in vitro* assays.^12^ Similarly, in our earlier work, 4 and 24 h inhalation of ultrafine carbon particles triggered an early stress response followed by a mild neutrophilic inflammation in the lungs of mice, with microarray analysis of lung homogenates uncovered induced expression of pro-inflammatory mediators including Lipocalin-2 (*Lcn2*), Osteopontin (*Spp1*), and Galectin-3 (*Lgals3*), which by RNA hybridization and antibody staining could be mapped to the alveolar epithelium (*Lcn2*) and alveolar macrophages (*Spp1*, *Lgals3*). Consequently, we suggested an early activation of both cell types as a major driver of this inflammatory response.^13^ Bulk transcriptomics has also been instrumental in linking nanomaterial responses to disease pathways. Nikota et al. employed comparative transcriptomic meta-analysis to assess toxicological outcomes of carbon black and CNT, comparing them with lung injury models such as bleomycin or bacterial infection. Their results indicated that CNT-induced signatures overlap with those of fibrogenic and infectious models, suggesting shared mechanisms of fibrosis initiation irrespective of CNT physicochemical differences.^14^ *In vitro* models have also benefited from transcriptomic profiling. For example, Tilton et al. analyzed transcriptomic changes in THP-1 macrophage-like cells exposed to MWCNT, identifying enrichment in cell proliferation, DNA repair, and Th17-related pathways.^15^ However, the translation of such *in vitro* signatures to *in vivo* outcomes remains challenging. Indeed, Kinaret et al. systematically compared transcriptomic profiles from THP-1 cells and mouse lung tissue after CNT exposure. While single-gene overlaps were minimal, network-level analyses revealed conserved molecular functions, emphasizing the role of intrinsic CNT properties in shaping responses and guiding the development of more predictive *in vitro* models.^16^ Although bulk transcriptomics has been highly informative, it remains limited by its averaging of gene expression across all cell types within a tissue. This masks cellular heterogeneity and can obscure the contributions of specific and sometimes rare cell populations that are critical for driving biological responses. Bulk data cannot resolve dynamic changes in immune cell influx, activation of cells into transient states, or the complex intercellular signaling crosstalk that orchestrates inflammatory responses.

In contrast, single-cell RNA sequencing (scRNA-seq) overcomes these limitations by enabling the high-resolution characterization of cellular heterogeneity and identification of activated or rare cell states. It also allows the inference of dynamic processes such as differentiation trajectories and cell–cell communication networks. While scRNA-seq is widely adopted in the biomedical field—for example, advancing our understanding of lung regeneration,^17^ SARS-CoV-2 pathogenesis, and the immune landscape during the COVID-19 pandemic^18–22^ —it has only recently begun to be applied to nanomaterial research and toxicology. Notably, Li et al. used scRNA-seq to investigate silica nanoparticle-induced pulmonary injury, uncovering interactions among epithelial cells, fibroblasts, and alveolar macrophages (AM), and identifying heat shock protein 1 (*Hsp1*) as a central driver of interstitial injury.^23^ Flores et al. applied scRNA-seq in a therapeutic context, showing that macrophage-targeting SWCNT-based nanotherapy modulates inflammatory gene expression in atherosclerotic lesions.^24^ Despite its strengths, scRNA-seq also carries limitations, including potential loss of fragile cell types during tissue dissociation, reduced sensitivity for low-abundance transcripts, and higher costs compared to bulk RNA-seq. Nonetheless, by capturing cell-type-specific perturbation signatures, scRNA-seq provides an unprecedented opportunity to identify the initiating cell populations and molecular pathways underlying CBN-induced pulmonary inflammation. With this approach, we can identify which cells initiate acute pulmonary inflammation and outline the transition into possible chronic inflammatory responses, subsequently causing tissue damage and dysfunction. Elucidating these cellular mechanisms is not only key to predicting adverse outcomes but also essential for identifying possible therapeutic targets to prevent exposure-related fibrosis or cancer development.

Here, we apply scRNA-seq to dissect lung responses to distinct CBN, thereby linking nanomaterial features to cell-specific responses. Specifically, we investigated spherical and soot-like carbon nanoparticles (CNP), flexible and tangled double-walled CNT (DWCNT), and rigid, needle-shaped multiwalled CNT (MWCNT), alongside lipopolysaccharide (LPS) as a positive control for acute inflammatory activation. This approach enabled us to capture nanomaterial-specific perturbations at single-cell resolution and to link CBN physicochemical features to cell-type-specific transcriptional responses. Our work delineates how shape and agglomeration state of CBN influence the initiation of pulmonary inflammation at the cellular level. To facilitate translation into predictive *in vitro* assays and more organotypic models, we further established a web-based ToxAtlas (https://organoidtox.shinyapps.io/nanoparticle_only_exposure_app/) for mapping CBN-specific transcriptional signatures. This resource represents a first step toward a comprehensive atlas of CBN-induced cellular responses, which is currently lacking in the field.

## Results and Discussion

### CBN-Specific Cell Response Patterns in the Lungs

Mice were exposed to CBN (physiochemical characteristics in Table 1) and LPS via intratracheal instillation to the lung for 12 h, and bronchoalveolar lavage (BAL) as well as lung histology was performed (Figure 1a). Doses of CBN (20 μg of CNP, 50 μg of DWCNT, and 15 μg of MWCNT) were chosen to elucidate the cellular initiation processes of pulmonary inflammation and therefore, to cause equal levels of pulmonary inflammation assessed by BAL neutrophil numbers at the early time point of 12 h, known to precede maximal levels at around 24 h. LPS was used as a positive control for acute inflammation and inflammatory macrophage activation (Figure 1b). Accordingly, alveolar neutrophil numbers assessed in BAL were equally elevated for all CBN without the presence of lymphocytes or multinucleated macrophages (Figures 1c and S1a). Alveolar clearance by phagocytosis was most evident for the spherical CNP, and the mass of all CBN localized to particle-laden alveolar macrophages (AM, Figures 1d and S1b), a considerable fraction of CNTs; however, remained on the epithelial surface, for DWCNT particularly as large agglomerates, but individual MWCNT could be visualized by darkfield imaging throughout the tissue. Particle-cell interaction is the first key event after pulmonary CBN deposition,^11^ and is accordingly described as a molecular initiating event of the inflammatory response in AOP #173: “pulmonary fibrosis” and #451: “lung cancer”.^11^ Here, scRNA-seq was performed to better understand cell- and CBN-specific mechanisms following exposure. We profiled 31,418 cells from 20 mouse lungs and identified 41 individual cell types/states, which can be classified into 9 major cell niches with highly CBN-specific cell response patterns (Table 2, Figures 1e–f and S1c,d). Differential gene expression (DGE) analysis revealed cell-type-specific expression changes in response to CBN (Figure 1g). Surprisingly, AM showed only little transcriptional activity despite the efficient uptake of CBN observed, which is contrasted by their strong response toward LPS. Cell types with stronger responses to CBN exposure as indicated by the DEG analysis included alveolar and airway epithelial cells, lipofibroblasts, and the endothelium. Enrichment analysis for hallmark signaling pathways throughout the lung indicated the importance of inflammatory gene expression changes in response to all CBN (Figure S1e), but mapping the HALLMARK_INFLAMMATORY_RESPONSE to the different cell niches uncovered CBN- and cell-specific response and frequency patterns (Figures 1h and S1c,d). Unlike known pathogen-associated molecular patterns (PAMPs), here LPS, the most powerful pro-inflammatory activator of macrophages, NP phagocytosing AM failed to show such signatures, demonstrating the discrimination between PAMP- and ‘sterile’ particle-induced inflammation cascades.^25^ Our results reveal highly CBN-specific, pro-inflammatory cell states that may initiate the infiltration by neutrophils. A similar approach to classify nanomaterial-specific inflammatory effects was carried out by Cho and colleagues.^26^ They investigated trends and types of acute and chronic inflammation after the instillation of various nanomaterials in rats by assessing BAL cell and protein composition. Inflammation was classified into so-called inflammatory footprints, lymphocytic (lymphocytes and IFN-γ), neutrophilic (neutrophils, CXCL2/MIP-2, and IL-1β), cytotoxic (LDH and total protein), as well as eosinophilic (eosinophils, CCL11/eotaxin, and IL-13) types, providing comprehensive information for nanomaterial safety assessment. The aim of our study was to better understand which lung cells initiate the inflammatory response in detail and untangle the underlying CBN-specific pro-inflammatory cell circuits.

**Figure 1:**
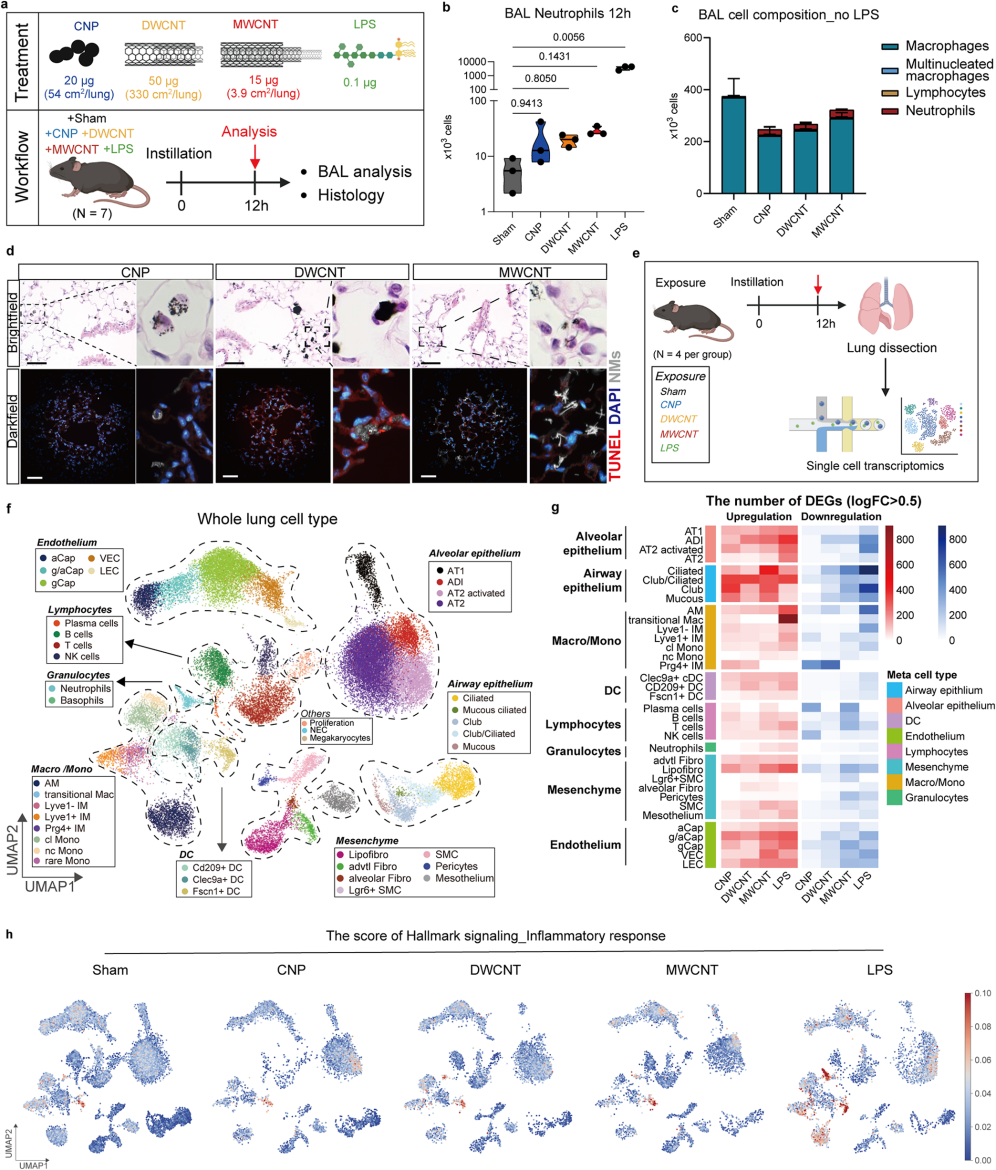
CBN-specific cell response patterns in the lungs (a). The experimental setup of the study. Mice (*n* = 7) were exposed to three different CBN (CNP: 20 μg, 54 cm^2^/lung; DWCNT: 50 μg, 330 cm^2^/lung; MWCNT: 15 μg, 3.9 cm^2^/lung), and LPS (0.1 μg/lung) and analyzed after 12 h. CBN doses generated comparable acute lung inflammation. Histology (*n* = 4) and bronchoalveolar lavage (BAL, *n* = 3) analyses were performed. (b). Differential neutrophil counts in BAL. (c). The characterization of different cell populations (macrophage, multinucleated macrophage, lymphocyte, and neutrophil) counts in BAL. (d). The engulfment of CBN by resident AM shown by brightfield microscopy and the cell–CBN interaction in mouse lungs shown by darkfield microscopy. Scale bar: 50 μm. (e). The experimental strategy of single-cell transcriptomic study. (f). Visualization of 41 annotated cell types and states in the mouse lungs by dimension-reduced single-cell transcriptomic data by Uniform Manifold Approximation and Projection (UMAP), classified into 9 major cell niches, which are listed in Table 2. (g). The effect size heatmap illustrates the overall number of significantly upregulated (red) and downregulated genes (blue). Differential gene expression (DGE) analysis was performed in each cell type and genes with log-fold change (logFC) > 0.5 or < −0.5 were counted. (h). The scoring of the signaling pathway “Hallmark_Inflammatory response” caused by different treatments. Data are shown as the mean ± standard error of the mean (SEM) of three mice (*n* = 4 for histology, *n* = 3 for BAL analysis), one-way ANOVA (nonparametric analysis; Kruskal–Wallis-Test) followed by Dunn’s multiple comparisons test was used for statistical analysis.

**Table 1:** Physiochemical Characteristics and Dispersion Quality of CBNs

CBN	CNP	DWCNT	MWCNT
provider (name)	degussa (Printex90)	nanocyl (NC2100)	hodogaya chemicals (Mitsui7)
material	carbon black	DWCNT	MWCNT
length (nm)		1000–10,000	5730 ± 491
diameter (nm)	14	3.5	74 ± 29
carbon (%)	99%	>90	98.1
BET (m^2^/g)	300	660	26
*Z*-average (nm)	347.9 ± 6.6	745 ± 15.5	3006.3 ± 357.8
PdI	0.348 ± 0.071	0.534 ± 0.050	0.206 ± 0.04

**Table 2:** Identified Cell Types and States in Murine Lungs After 12 h of CBN Instillation

major cell niches	cell type/state	abbreviation
alveolar epithelium	alveolar epithelial type I cells	AT1
	alveolar epithelial type II cells	AT2
	alveolar differentiation intermediate cells	ADI
	alveolar epithelial type II cells, activated	AT2 activated
airway epithelium	ciliated cells	Ciliated
	mucous ciliated cells	Mucous ciliated
	club cells	Club
	club/ciliated cells	Club/Ciliated
	mucous cells	Mucous
	neuroendocrine cells	NEC
monocytes/macrophages	alveolar macrophages	AM
	transitional macrophages	Transitional Mac
	Lyve1- interstitial macrophages	Lyve1- IM
	Lyve1+ interstitial macrophages	Lyve1+ IM
	Prg4+ interstitial macrophages	Prg4+ IM
	classical monocytes	cl Mono
	nonclassical monocytes	nc Mono
	rare monocytes	rare Mono
dendritic cells	Cd209+ dendritic cells	Cd209+ DC
	C-Type lectin Clec9a+ dendritic cells	Clec9a+ DC
	fascin actin-bundling protein 1 (FSCN1)+ dendritic cells	Fscn1+ DC
bone marrow-derived cells	megakaryocytes	Megakaryocytes
lymphocytes	plasma cells	Plasma cells
	B lymphocytes	B cells
	T lymphocytes	T cells
	natural killer cells	NK cells
granulocytes	neutrophils	Neutrophils
	basophils	Basophils
mesenchyme (mesenchymal connective tissue)	lipofibroblasts	Lipofibro/LF
	adventitial fibroblasts	advtl Fibro
	alveolar fibroblasts	alveolar Fibro
	leucine rich repeat containing G protein-coupled receptor 6 (Lgr6)+ smooth muscle cells	Lgr6+ SMC
	smooth muscle cells	SMC
	pericytes	Pericytes
	mesothelium	Mesothelium
endothelium	aerocytes, alveolar capillary endothelial cells	aCap
	intermediate capillary endothelial cells	g/aCap
	general capillary endothelial cells	gCap
	Vcam1+ endothelial cells	VEC
	lymphatic endothelial cells	LEC
**-**	proliferating cells	Proliferation

### CBN-Specific Cell Circuits Causing Pro-Inflammatory Responses

In an initial step, cell states (within the epithelial, fibroblast, and macrophage lineages) with possibly pro-inflammatory functions and observed CBN-specific inflammatory hallmark scores (Figure 2a–d) were analyzed. The endothelial compartment showed little contribution to the gene regulation of the early inflammatory response and was hence excluded (Figure S1g,h). Gene expression signatures of interest can be studied using our ToxAtlas: https://organoidtox.shinyapps.io/nanoparticle_only_exposure_app/. Examples of *Cxcl1* expression, or *Cxcl1* and *Csf2* coexpression in alveolar epithelial type II cells (AT2), as well as dot plots illustrating the CBN-induced genes *Cxcl1, Csf2, Ccl2, – 3, – 11*, and 19 are provided in the **ToxAtlas webtool tutorial**. Pulmonary CNP exposure acted most notably on AT2 cells, causing an activation of AT2 (AT2 activated) and to some extent lipofibroblasts, as shown by increased expression of inflammatory gene sets in these cells (Figure 2a–d).

**Figure 2:**
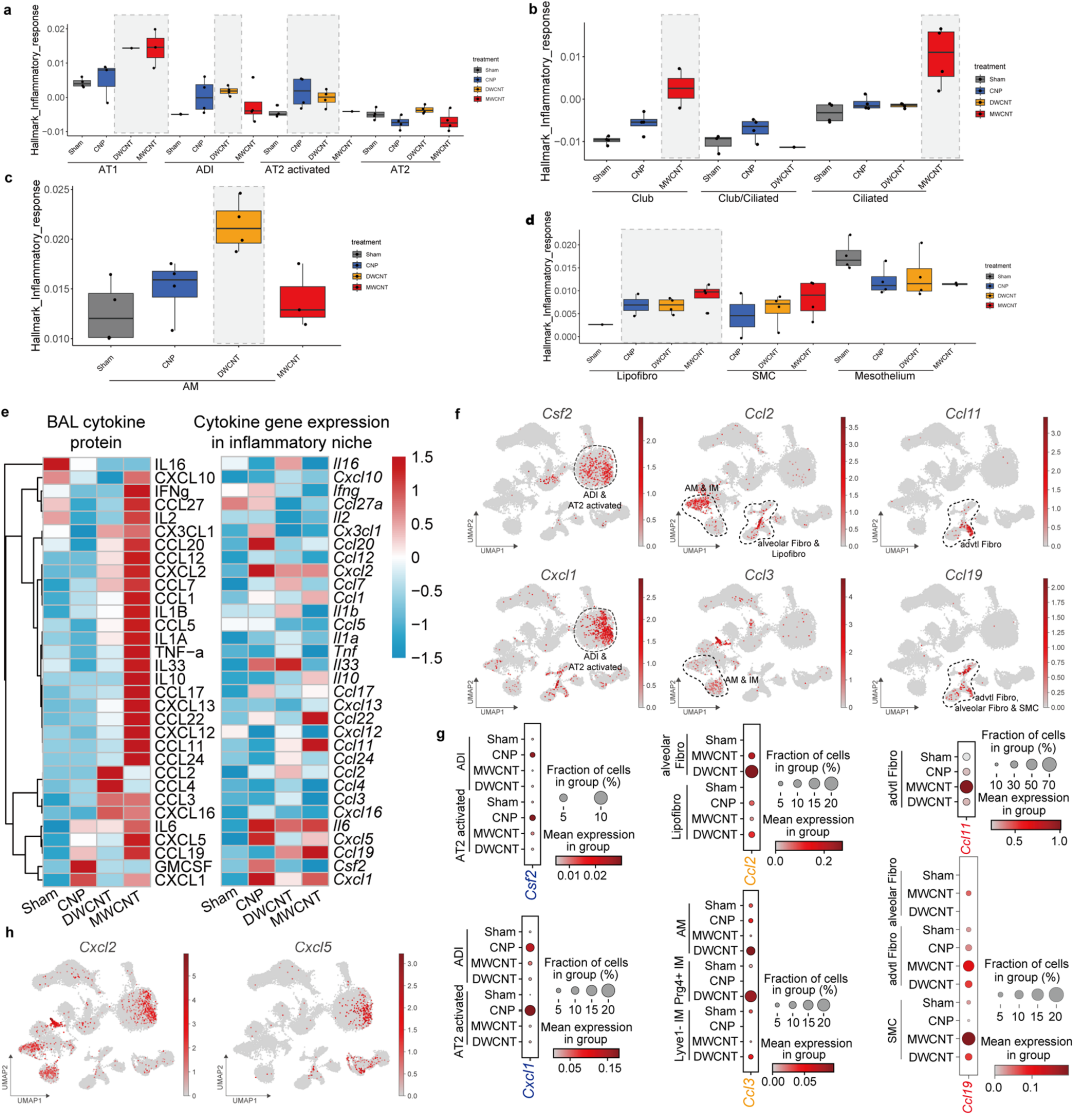
Cell mapping of the CBN-specific course of pro-inflammatory cytokines. The scoring of the “pro-inflammatory response” pathway exhibited CBN-specific patterns in alveolar epithelial cell types (a), airway epithelial cells (b), macrophage cell types (c) and also mesenchymal cell types (d). (e). Heatmap of BAL cytokine protein level compared to relative mRNA level in defined “inflammatory niche” including alveolar and airway epithelial cells, macrophages, and mesenchymal cells. (f). The UMAPs of CBN-specific cytokines, two identified cytokines were shown for each CBN, the localization of each cytokine in cell types is circled and labeled on the plots. (g). The dotplot of specific cytokine gene induction caused by different CBN in different cell types. (h). UMAP of *Cxcl2* and *Cxcl5*. Experiments were performed as *n* = 3 for BAL cytokine measurements, *n* = 4 for all transcriptomics analysis. One-way ANOVA (nonparametric analysis; Kruskal–Wallis Test) followed by Dunn’s multiple comparisons test was used for statistical analysis.

In contrast, for both CNT, elevated expression of inflammation hallmark genes was found in alveolar epithelial type I cells (AT1), indicating a direct interaction of the larger agglomerate-forming, fiber-shaped CBN with the respiratory barrier. Furthermore, for DWCNT we observed upregulated hallmark genes in the AT2-derived cell states of alveolar differentiation intermediates and AT2-activated cells (Figure 2a–d), whereas only MWCNT caused strong changes in club and ciliated cells. Notably, a clear inflammatory activation of AM was only detected upon DWCNT exposure (Figure 2c). In the underlying mesenchymal niche, lipofibroblasts showed an early pro-inflammatory response after all CBN exposures (Figure 2d). To better understand the initiation of airspace neutrophilia, cell type and CBN-specific cytokine expression patterns that may affect the recruitment of leukocytes were delineated (Figures 2e–g and S2).

The cytokines detected in the airspace and ‘inflammatory niche’ validated the highly CBN-specific pattern observed on gene expression level, with the strongest response observable for MWCNT-treated lungs, therefore reflecting the well-described toxicity of MWCNT.^27^ CNP exposure specifically elevated neutrophil chemotactic CXCL1 and GM-CSF (encoded by *Csf2*) expression in AT2-activated cells. DWCNT in contrast elicited monocyte chemotactic CCL2 in fibroblasts and monocyte and neutrophil attractant CCL3 in AM and interstitial macrophages (IM). MWCNT induced the Th2 cytokine CCL11, chemotactic for neutrophils, monocytes, and particularly for eosinophils, in adventitial fibroblasts as well as CCL19, another Th2 cytokine, attracting lymphocytes and dendritic cells in adventitial fibroblasts and smooth muscle cells. The neutrophil chemoattractants CXCL2 and *–*5 were noticeably released upon MWCNT, but these BAL levels did not match the transcription profile (Figures 2h and S2c). Overall, our analyses indicate no evident role of the bronchial epithelium in early cytokine release (Figure 2f,h). In summary, the three CBN-triggered striking material-specific gene expression signatures, validated by cytokine release patterns in the airspace, underscoring CBN-specific modes of action for initiation of inflammation (Figure 2g). This study thereby highlights the importance of deciphering CBN-initiated inflammation at the cell type level for a mechanistic understanding of CBN hazard and *in vitro* testing approaches.

### Epithelial Involvement for CNP-Specific Cell Perturbations

After successfully identifying pro-inflammatory cell circuits crucial to the initiation of airspace neutrophilia for each CBN, contributing respective cell niches were investigated in more detail. Since CNP-driven neutrophil chemoattractant release was to a large extent orchestrated by AT2 activation (Figure 2g), we focused on the epithelial niche first (Figures 3a and S3a,b). Relative cell frequency analyses indicated an acute accumulation of AT2-activated cells (*Lcn2, Il33, and Lrg1*) for CNP and DWCNT exposure (Figure 3b,c), thereby increasing the expression of pro-inflammatory genes in the alveolar epithelium. Only MWCNT induced a distinct state of Krt8+ alveolar differentiation intermediates, known for their importance in epithelial regeneration.^17^ Alveolar differentiation intermediates (characterized by genes such as *Krt8, Ctsh, Ezr*) represent a transitional alveolar cell state preceding terminal differentiation of AT1 cells that emerges in many mouse lung injury models.^17^

**Figure 3:**
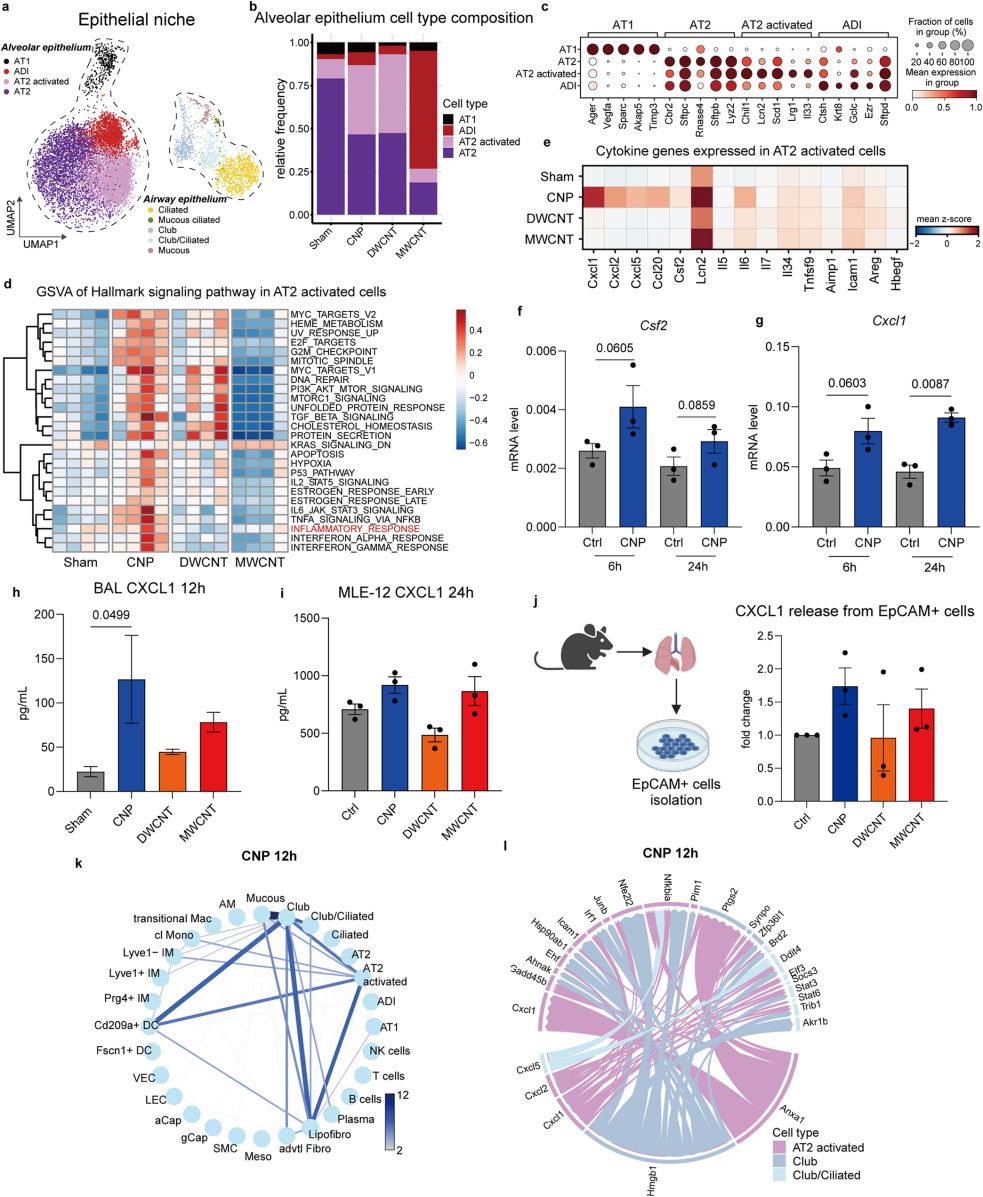
CNP exposure caused neutrophil attraction and formed cellular communications within the local alveolar environment (a). UMAP embedding illustrates cell types and states in the epithelial niche. (b). Treatment-dependent alteration of cell relative frequencies with activation of AT2 (AT2 activated) for CNP and DWCNT and alveolar differentiation intermediates upon MWCNT exposure. (c). Dotplots displaying the top 5 marker genes for each annotated cell type. (d). Heatmap of gene set variation analysis of hallmark signaling pathways on AT2-activated cells. (e). Matrixplot of pro-inflammatory cytokine gene expression caused by different CBN in AT2-activated cells. (f). *Csf2* induction by CNP exposure in MLE12 cells after 6 and 24 h measured by qPCR. (g). *Cxcl1* induction by CNP exposure in MLE12 cells after 6 and 24 h measured by qPCR. In (f, g), data are shown as mean ± SEM (*n* = 3). For each time point, a Student *t* test was performed between two groups. *P* value was shown and *P* value <0.05 was considered statistically significant. (h). BAL CXCL1 protein level measured by ELISA. (i). The CXCL1 release into MLE-12 cell supernatant exposed to different CBN after 24 h. (j). Mouse primary epithelial cells (EpCAM+ cells) isolation and CXCL1 release into supernatants induced by different CBN. (k). Connectomes based on induced differential gene expression (DGE) analysis (treatment vs sham) show computationally inferred cellular communication strength in response to CNP. For each circle plot, edge weight and color represent the number of ligand–receptor pairs between interacting cell types. (l). The visualization of NicheNet analysis by circosplot illustrates the connectome of the situation before in (j) described prevailing interaction between different epithelial cells upon CNP exposure. The lower part of the circosplot is identified as “sender” cell types whereas the upper part draws the “receiver” cell types, signaling interaction by different genes were shown. For (h–j), data are shown as the mean ± SEM (*n* = 3), one-way ANOVA followed by Dunn’s multiple comparisons test was used for statistical analysis.

Gene set variation analysis (GSVA) revealed a pronounced pro-inflammatory response signaling in CNP-treated lungs, depicted by IL6-JAK-STAT3, interferon, and TNFa signaling (Figure 3d). While this was in contrast to MWCNT, CNP pathways overlapped with DWCNT for protein secretion, DNA repair, and cell proliferation and showed similar levels of AT2 activated cells. Profiling cytokine expression within the gene sets upregulated in the AT2-activated cells indicated that the pro-inflammatory response specific for CNP is mainly driven by strong activation of epithelial neutrophil attractants (*Cxcl1*, *–*5, *Ccl20, Csf2, Il6*) (Figure 3e). To reveal whether the described pro-inflammatory AT2 response (Figure 2e,f) was directly triggered by particle–cell interaction, murine AT2-like MLE12 cells were exposed to the three CBN but detected only moderate levels of *Cxcl1* and *Csf2* induction by CNP treatment (Figure 3f,g). However, looking into cytokine levels released into the medium of MLE-12 or murine primary lung epithelial cells (EpCAM+ cells) revealed a similar CXCL1 response pattern for the three CBN (Figure 3h–j) as detected in BAL. Notably, TNF but not IL1 signaling was specifically induced in CNP-stimulated AT2 cells (Figure 3d), and TNF-α used as *in vitro* positive control induced high expression of *Csf2*, in contrast to MyD88 dependent LPS or IL1α, thereby supporting the previously described hypothesis of locally released TNF-α by CBN-phagocytosing AMs tipping off the neutrophil influx in the vicinity to CBN deposition (Figure S3c,d).^28^ However, additional cell–cell interactions might be required for effective AT2 cell stimulation. Ligand–receptor pairings-based connectome analysis showed a responsive involvement of AT2 activated cells with the bronchiolar niche as well as with inflammatory, monocyte-derived dendritic cells (CD209+ DCs) (Figure 3k). Interestingly, many connections were found at this acute time point with lipofibroblasts, structurally located near AT2s in the alveolar barrier for functional support, and therefore, eventually also active during the inflammatory response. Intercellular communication analysis by NicheNet revealed a pro-inflammatory cell circuit *via Anxa1, Hmgb1, Cxcl1, −2, −5*, toward *Cxcl1, Icam1, Nfkbia, Stat*3, *Ptgs2/Cox2*, etc. (Figure 3l). A direct stimulation from damaged AT2 cells via ANXA1 as suggested, however, could not be supported for its pro-inflammatory interepithelial communication loop in our MLE12 *in vitro* model (Figure S3f). Even though we and others have previously shown that carbon nanoparticles can trigger oxidative stress to lung cells,^29,30^ GSVA pathway profiling did not provide evidence for oxidative stress to be specifically triggered here. Nevertheless, the activation of AT2 cells by CNP-cell interaction, triggering *Csf2* and *Cxcl1* gene expression and release into the air space seems to represent a crucial signaling loop for the key event, neutrophil attraction. This process might be supported by further actions such as AM released local TNF-α or CCL20 release from the adjacent epithelium enforcing the pro-inflammatory environment. CNP therefore develops its inflammatory potency by activating AT2, triggering pro-inflammatory signaling pathways (*e.g*., IL6-JAK-STAT3, TNF-α), causing neutrophil chemoattractant release (e.g., CXCL1, GM-CSF).

### CNT Exposure Induces Alveolar Damage and DAMP Release

Pulmonary exposure to CNTs caused cell damage to the alveolar barrier, indicated by protein exudation (Figure 4a) and epithelial TUNEL-positive cells (Figure 4b,c), particularly for DWCNT exposure (Figure 4d). Further investigations revealed that the DNA-damage (TUNEL) signal predominantly localized to AT1 and AT2 by double-staining with AQP5 and pro-SPC separately (Figure 4d–f), whereas an elevated damage-associated molecular pattern (DAMP) expression score (Figures 4g and S4a), mostly based on *Il*33 (Figure 4h) was observed for all CBN. In contrast to its mRNA expression, IL-33 protein, the epithelial alarmin and DAMP, which at steady state accumulates in the nucleus, was released into the lining fluid, specifically in response to MWCNT-mediated injury (Figure 4i). Similar release patterns *in vitro* were recreated by exposing the murine epithelial AT2-like cell line (LA-4) to MWCNT (Figures 4j and S4b,c). The peculiarity of the MWCNT-related cell injury is in concert with the induction of a unique alveolar differentiation intermediate signature in the alveolar epithelium, only observed upon MWCNT treatment and indicating the regenerative processes after CNT injury (Figure 3b), which if chronic, are discussed as key events for fibrosis.^31^ The pattern of IL-33 release from MWCNT-damaged epithelial cells was also reported by Beamer et al. for the 24 h time point and illustrated an IL-33-ST2 axis in MWCNT induced eosinophil recruitment, with Th2-associated pulmonary inflammation.^32^ With their work, Katwa and colleagues highlighted the relevance of mast cells for the IL-33-ST2 axis upon MWCNT exposure which orchestrates adverse pulmonary and cardiovascular responses.^33^ As a consequence, reduced pulmonary inflammation and injury were observed in mast cell–deficient mice, as well as in IL-33 receptor–deficient (ST2^–^/^–^) mice, following MWCNT instillation compared to wild-type mice.^34^ In contrast to MWCNT, CNP stimulated the epithelium only toward an acute proinflammatory response (Figure 3), without detectable cell damage or DAMP release. The next step was to understand the contribution of resident AM to the detected cell and tissue damage. TUNEL signals in CD11c+ macrophages, i.e., AM, were significantly elevated for DWCNT-treated lungs (Figure 4k,l). Elevated BAL levels of another alarmin, IL1α, but not inflammasome-dependent IL1β, were detected upon CNT and particularly MWCNT exposure (Figures 4m and S4d,e). *Il33* is mostly expressed by AT2 cells (Figure S2a), whereas *Il1a* is very abundant in AMs, suggesting macrophage injury upon CNT uptake (Figure 4n). Accordingly, treatment of primary macrophages with CBN caused IL1α release at cytotoxic doses of MWCNT (Figure 4o). Interestingly, IL-1R1-deficient mice exhibited impaired acute pulmonary inflammation upon MWCNT exposure, as shown in several studies underpinning its relevance for the induction of inflammation also during sterile inflammation.^35,36^ The early release of IL1α from silica or MWCNT exposed AM into the airspace of mice has been elegantly described by Huaux and co-workers to precede lung Il1β expression and neutrophilia, suggesting IL1α as a master cytokine and predictor of acute, particle-triggered inflammation in nanotoxicological studies.^37^ In addition to IL1α, BAL cytokine profiling revealed the release of CCL2, *–*3 and *–*4 into the airspace after DWCNT exposure (Figure 2e), specifically mapping *Ccl2* and *–3* to resident lung macrophages (Figure 2f). The hypothesis that direct particle–cell interactions are sufficient to stimulate DWCNT-induced lung macrophage activation and cytokine expression could not be supported at the *in vitro* level. DWCNT also previously failed to trigger a proinflammatory signature in a murine model of bone marrow-derived macrophages (Ana-1 cell line) *in vitro*, even though the MAP kinase p38 was activated in a particle-dependent manner.^30^ However, CNT exposure of the malignant ascites-derived macrophage line, J774.1, indicated induced *Ccl2* levels 24 h after DWCNT treatment, with even higher effects for *Ccl2* and *–3* after MWCNT (Figures 4p,q and S4f). Together, our data suggest alarmin release from damaged epithelial cells, for DWCNT, and from damaged macrophages for MWCNT, to drive CNT-induced lung inflammation. A typical pro-inflammatory macrophage activation as observed upon stimulation by pathogens such as LPS was not observed.

**Figure 4:**
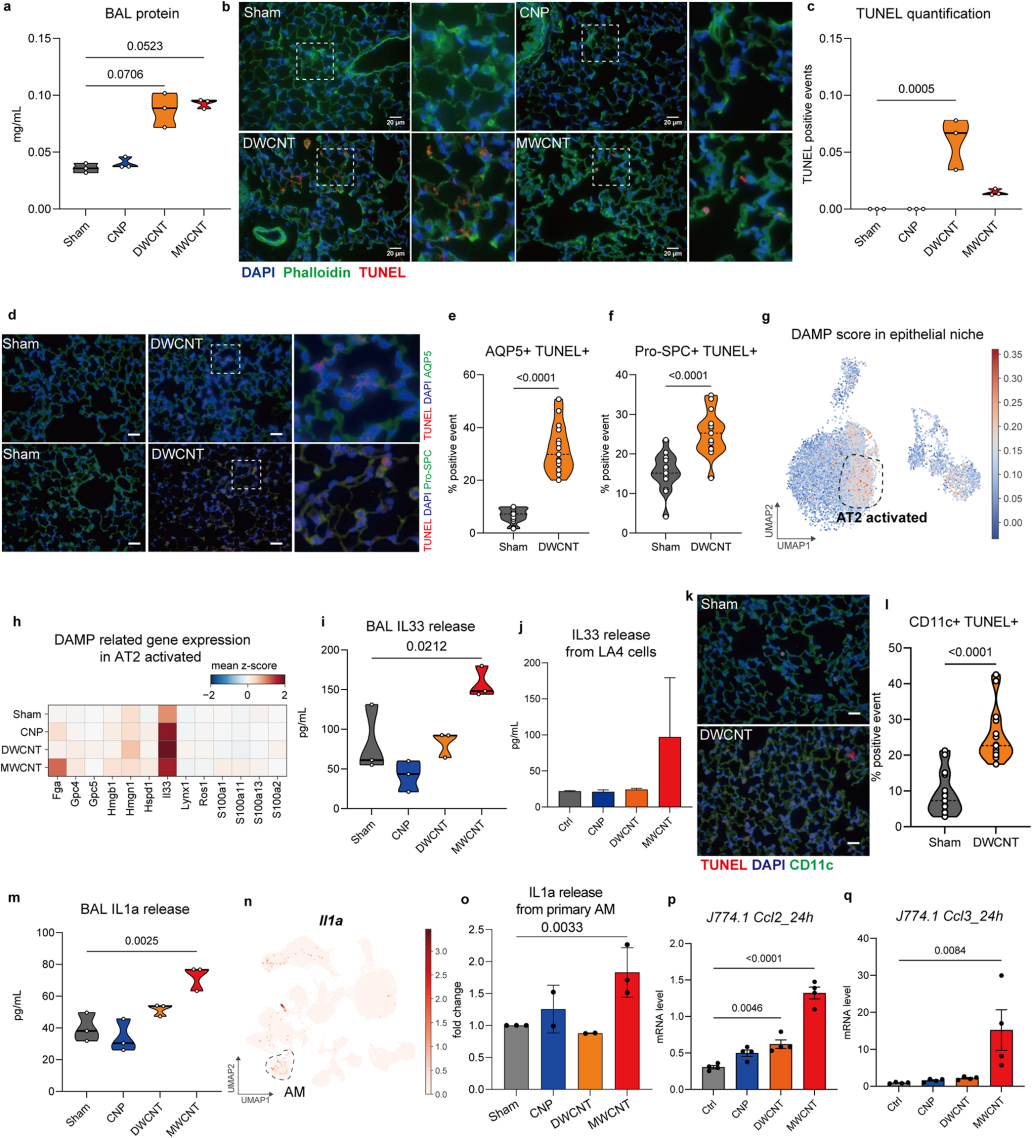
CNT exposure causes acute epithelial damage and DAMP release (a). BAL total protein levels detected after CBN treatment as a measurement for epithelial damage. (b). TUNEL assay of the DNA damage caused by different CBN. DAPI: blue, Phalloidin 488: green, TUNEL: red. Scale bar: 20 μm. (c). The quantification of TUNEL-positive events in mouse lung tissue at 12 h (*n* = 3). (d). Double staining of TUNEL-positive cells in AT1 and AT2 cells marked by AQP5 and pro-SPC. DAPI: blue, AQP5 or pro-SPC: green, TUNEL: red. Scale bar: 20 μm. The quantification of TUNEL & APQ5 double positive events (e) and TUNEL & APQ5 double positive events (f) in mouse lung tissue at 12 h. (g). UMAP displays density and distribution of cells showing a high DAMP score in epithelial niche. (h). A matrixplot of DAMP-related gene expression caused by different CBN in AT2-activated cells. (i). IL-33 release caused by CBN into BAL fluid measured by ELISA. (j). IL-33 release into the supernatant of mouse AT2-like cells LA4 caused by CBN measured by ELISA. (k). Double staining of TUNEL-positive cells in AMs marked by CD11c. DAPI: blue, CD11c: green, TUNEL: red. Scale bar: 20 μm. (l). The quantification of TUNEL & CD11c double positive events in mouse lung tissue at 12 h. (m). IL1α release caused by CBN into BAL fluid measured by ELISA. (n). The visualization of *Il1a* expression and localization by UMAP. (o). IL1α release upon CBN exposure in isolated mouse primary AMs. The expression of *Ccl2* (p) and *Ccl3* (q) induced by CBN exposure in AM-like J774.1 cells. CNP (50 μg/mL), DWCNT (50 μg/mL), and MWCNT (30 μg/mL) were used in the *in vitro* study. Data were shown as the mean ± SEM (*n* = 3 or 4), one-way ANOVA followed by Dunn’s multiple comparisons test was used for statistical analysis.

### Mesenchymal Activation Drives CBN-Induced Acute Inflammation

As identified by our cytokine mapping approach (Figure 2d,f,g), the mesenchymal niche, and here particularly lipofibroblasts, profoundly contribute to the earliest CBN-induced inflammatory responses. *Ccl2* was mapped to alveolar fibroblasts and lipofibroblasts after DWCNT exposure and *Ccl11* was induced in adventitial fibroblasts as well as *Ccl19* in smooth muscle cells and adventitial fibroblasts after MWCNT exposure, respectively. Single-cell transcriptomics identified 7 individual mesenchymal cell clusters, with lipofibroblasts being the most abundant fibroblast cell type (Figures 5a,b and S5a). As mentioned before (Figure 2d) lipofibroblasts featured an upregulated gene set in the hallmark pro-inflammatory response for all CBN tested. For MWCNT, this pro-inflammatory signature was particularly supported by the activation of TNFa signaling via NF-kB (Figure 5c). The connectome analysis highlighted a pronounced involvement of lipofibroblasts together with pulmonary epithelial cells for CNP-treated lungs (Figure 2j). For DWCNT, the mesenchymal communication shifted to alveolar differentiation intermediates, suggesting a crosstalk of injured alveolar epithelial cells to the mesenchyme (lipofibroblasts and smooth muscle cells) (Figure 5d). For MWCNT the mesenchymal communication focused to adventitial fibroblasts with bronchial epithelial cells (Figure 5e). Taking previous results into account, i.e., signatures for inflammation (Figure 2) and DAMP signaling (Figure 4), in combination with the observed cellular crosstalk in the induced connectomes (Figure 5), indicate the following CBN-triggered communications: AT2 cells get activated by CNP exposure to interact with lipofibroblasts, by which the mesenchyme acquires a unique pro-inflammatory signature with high *Il33*, *Ccl20, −*22 and *27a* (Figure 5f). Furthermore, lipofibroblasts acquire a monocyte-attracting signature upon DWCNT exposure to the lung (*Ccl2, Ccl7;* Figure 5f), subsequently recruiting monocytes labeled by CCR2 into the interstitium (Figure S5b). MWCNT, in contrast, damage the bronchiolar epithelium, and injure ciliated cells, stimulate lipofibroblasts and adventitial fibroblasts, due to which the mesenchyme acquires a Th2 cytokine signature (*Ccl11, Ccl19*), matching the eosinophilia observed upon MWCNT exposure.^38^ While all treatments cause a signature for damage response fibroblast (*Cxcl1, Il6*),^39^ the mesenchymal niche seems to act as a switch point shaping inflammation, damage and injury signals toward transient neutrophilia, or monocyte and Th2 cell recruitment. Direct *in vitro* stimulation of fibroblasts with the three CBN did not show any pro-inflammatory activation (Figure 5g,h), which, however, was generated by IL1α or TNFα cytokine treatment (Figure S5c,d), supporting the requirement of paracrine cell–cell interactions. Noteworthy is the alarmin IL1α, which accumulated in BAL upon MWCNT treatment (Figure 4m) and was shown to be released from MWCNT-damaged AMs (Figure 4o), effectively inducing *Ccl11* expression in CCL-206 fibroblast cells (Figure S5d), presenting the central axis of the macrophage-fibroblast circuit. On the other hand, the release of IL-33, specifically from MWCNT exposed epithelial cells (Figure 4i,j), could also contribute to the transcriptional activation of *Ccl11*, as was recently described.^40^ IL-33-stimulation and release might generate the Th2 milieu, and in synergy with the mesenchymal Th2 cytokines identified (Figure 5f), including CCL11 and *–*19 (Figure 2g), shape the environment for the profibrotic toxicity of MWCNT.^41^ Together our data suggests fibroblasts to be not merely effector cells during the manifestation of the fibrotic responses to inhaled fibers but also to present the interstitial hub crucial in contributing to the local pro-inflammatory and potentially pro-fibrotic cytokine milieu.

**Figure 5:**
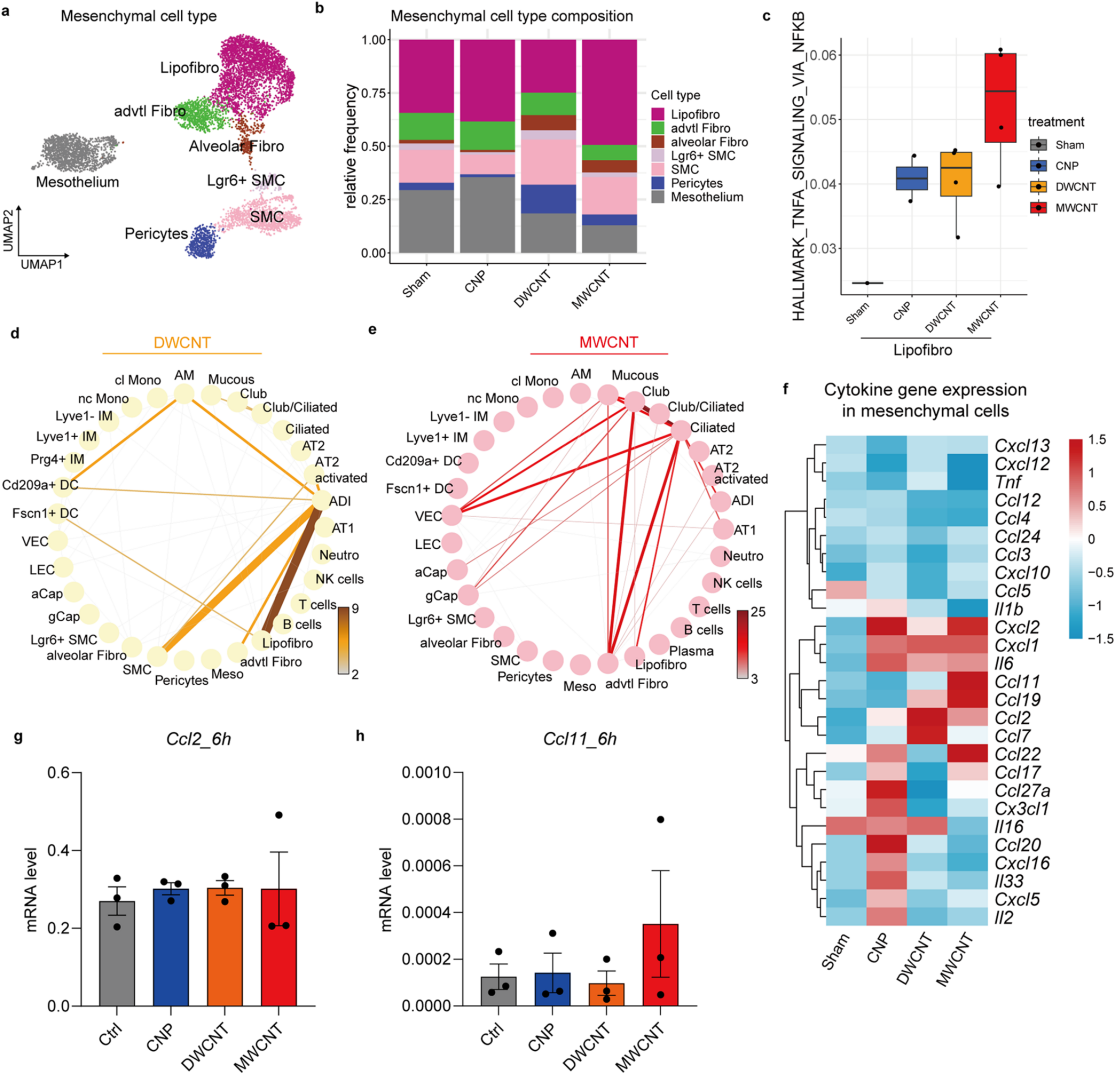
Mesenchymal activation supports CBN-induced acute inflammation (a). UMAP embedding illustrates cell type identity for the mesenchyme niche. (b). Relative cell frequency shows treatment-dependent effects, and lipofibroblasts (Lipofibro) are the most abundant fibroblast cell types. (c). Boxplot of treatment-dependent activation of the hallmark signaling pathway “TNF-a signaling via NFKB” in lipofibroblasts. Connectomes based on induced differential gene expression (DGE) analysis (treatment vs sham) show computationally inferred cellular communication strength in response to DWCNT (d) and MWCNT (e). For each circle plot, edge weight and color represent the number of ligand–receptor pairs between interacting cell types. (f). Heatmap of cytokine gene expression related to pro-inflammatory response in mesenchyme. The expression of *Ccl2* (g) and *Ccl11* (h) by CBN exposure in CCL-206 cells after 6 measured by qPCR. CNP (50 μg/mL), DWCNT (50 μg/mL) and MWCNT (30 μg/mL) were used in the *in vitro* study. For *in vitro* experiments, data are shown as mean ± SEM (*n* = 3), one-way ANOVA followed by Dunn’s multiple comparisons test was used for statistical analysis.

### Limitations of the Study

This study sheds new light on the early material-specific cell responses leading to sterile inflammation after CBN inhalation using innovative transcriptomics analyses at single-cell resolution, yet several limitations must be acknowledged. The current investigations were limited to one dose and time point of acute exposure to three different-shaped CBN, namely, CNP, DWCNT, and MWCNT which do not represent all inhalable CBN forms. It is therefore difficult to extrapolate these results to other materials of varying purity, agglomeration state, length, aspect ratio, or even to two-dimensional (2D) materials like graphene.^42^ Nevertheless, our study indicates that carbon black-like spherical particles have minimal impact on macrophages while CNT-shaped CBN can initiate pro-inflammatory activation of AM. Moreover, because of limited availability of cell type-specific cell lines or knowledge of physiological culture conditions to fully support primary niche cells (*e.g*. AT1, lipofibroblasts, etc.), the *in vitro* replication of *in vivo* observed response patterns faces commonly known challenges. This is in addition to expected differences when comparing complex, multicellular, and multiorgan *in vivo* conditions (*e.g*. immune system, blood flow, lung lining fluid and breathing movement) to simplified *in vitro* results. Nevertheless, insights about respective cell types and states involved in the initiation of CBM triggered inflammatory pathways, common nanotoxicological key events, provide a necessary foundation for the design of future *in vitro* safety assessment approaches. The limitations of available cell lines, primary cells, and this overall strategy have been evident in our *in vitro* experiments, yet they are integral and important data to share with the community and motivate recent efforts to develop mouse and human lung organoids for hazard assessment.^43^

## Conclusions

Leveraging the power of scRNA-seq, this study provides critical insights into the cell-specific mechanisms underlying the initiation of inflammation to CBN revealing how distinct material properties drive unique molecular and cellular perturbations. In this study, we investigated chemically similar yet physically distinct CBN to gain deeper insights into how the particle shape and agglomeration state influence the initiation of inflammatory responses following pulmonary deposition. CBN were chosen due to their structural diversity and negligible solubility in biological media, classifying them as poorly soluble particles of low inherent toxicity. This stands in sharp contrast to certain metal oxide nanomaterials such as ZnO, NiO, or CuO, which display high cytotoxicity primarily through endosomal and lysosomal ion release upon cellular uptake.^26,44^ In comparison, the mechanisms of action of CBN are far less well-defined, as is their capacity to induce acute or chronic inflammatory responses in the lung.

Even though chemically similar, CBN initiate pulmonary inflammation of varying quality via distinct MoA in lung resident epithelial cells, fibroblasts and macrophages (Figure 6). Spherical CNP triggered lung inflammation via proinflammatory activation of the alveolar epithelium to release neutrophil chemoattractants without the apparent involvement of damage signaling, cell death, or transcriptional stimulation of resident lung macrophages in vivo. The cytokine genes for neutrophil attractants *Cxcl1* and *Csf2* induced in AT2 cells as key event markers could be supported at the *in vitro* level, although their moderate levels of induction by direct particle–cell interaction point to involvement of additional factors, such as the interaction with fibroblasts, or the local release of proinflammatory mediators from CBN-phagocytosing AMs.^28^ In contrast to the oxidative stress paradigm proposed in previous studies, which implicates the oxidative surface properties of CBN as a key driver of acute lung inflammation, our pathway analysis did not support the involvement of ROS sensing, and genes associated with the HALLMARK_REACTIVE_OXYGEN_SPECIES_PATHWAY were not significantly enriched.^45–49^ Both CNT, in contrast to CNP, caused cell damage and death signatures with subsequent alarmin release *in vivo* and *in vitro*, especially observed for the rigid MWCNT, known for their respiratory fiber toxicity. MWCNT exposure was dominated by alarmin release, IL1α from injured macrophages and IL-33 from the epithelium, both demonstrated in our minimal cell exposure models. A similar IL1α-based mode of action had been described for crystalline silica before.^37^ For the DWCNT with a less rigid structure and curled agglomeration, we observed cytotoxicity and cell damage specific to the fragile alveolar epithelium, associated with pro-inflammatory macrophage and lipofibroblast activation and related cytokine release into the airspace (CCL2 and *–*3). Underlying gene expression patterns (*Ccl2* and *–*3) could be replicated in a macrophage cell line *in vitro*. DWCNT further caused the fibroblast niche to express the monocyte macrophage chemoattractants *Ccl2 and −*7, promoting interstitial attraction of mononuclear cells, as was previously described.^30^ In line with that, *Ccl2* expression was upregulated in a lung fibroblast cell line upon DWCNT exposure. Because of the lack of directly MWCNT-induced Th2 cytokine release from *in vitro* treated fibroblast, we propose that alarmins released from damaged cells rather than direct CNT mesenchymal interaction, drive the fate of CNT-induced tissue inflammation, and act in concert to induce a Th2 environment in activated fibroblasts which later might recruit lymphocytes and eosinophils, thereby self-perpetuating a type 2 inflammatory, pro-fibrotic feedback loop.^32,50,51^ Here, MWCNT-associated epithelial release of IL-33 might play a decisive role as described by Beamer and colleagues.^32^ While the respiratory epithelial cell sheet is well-known as the first line of defense and thus the prime candidate for the initiation of the inflammatory defense against inhaled particles, our study identified a new role for the underlying mesenchyme and, here in particular, the AT2 adjacent lipofibroblasts. Our data underlines the interplay of structural cells, such as fibroblasts and epithelial cells, by sensing macrophage damage to function as key regulators of the pulmonary innate immune response, provoked by inhaled CBN. Finally, our web-based ToxAtlas (https://organoidtox.shinyapps.io/nanoparticle_only_exposure_app/) serves as an initial resource to map CBN-specific gene expression patterns, offering a valuable tool to the field for precision *in vitro* testing and the rational design of safer nanomaterials. Future expansions of the webtool will integrate additional scRNA-seq data sets of supplementary CBN and later time points to strengthen its utility for mapping CBN-induced responses.

**Figure 6:**
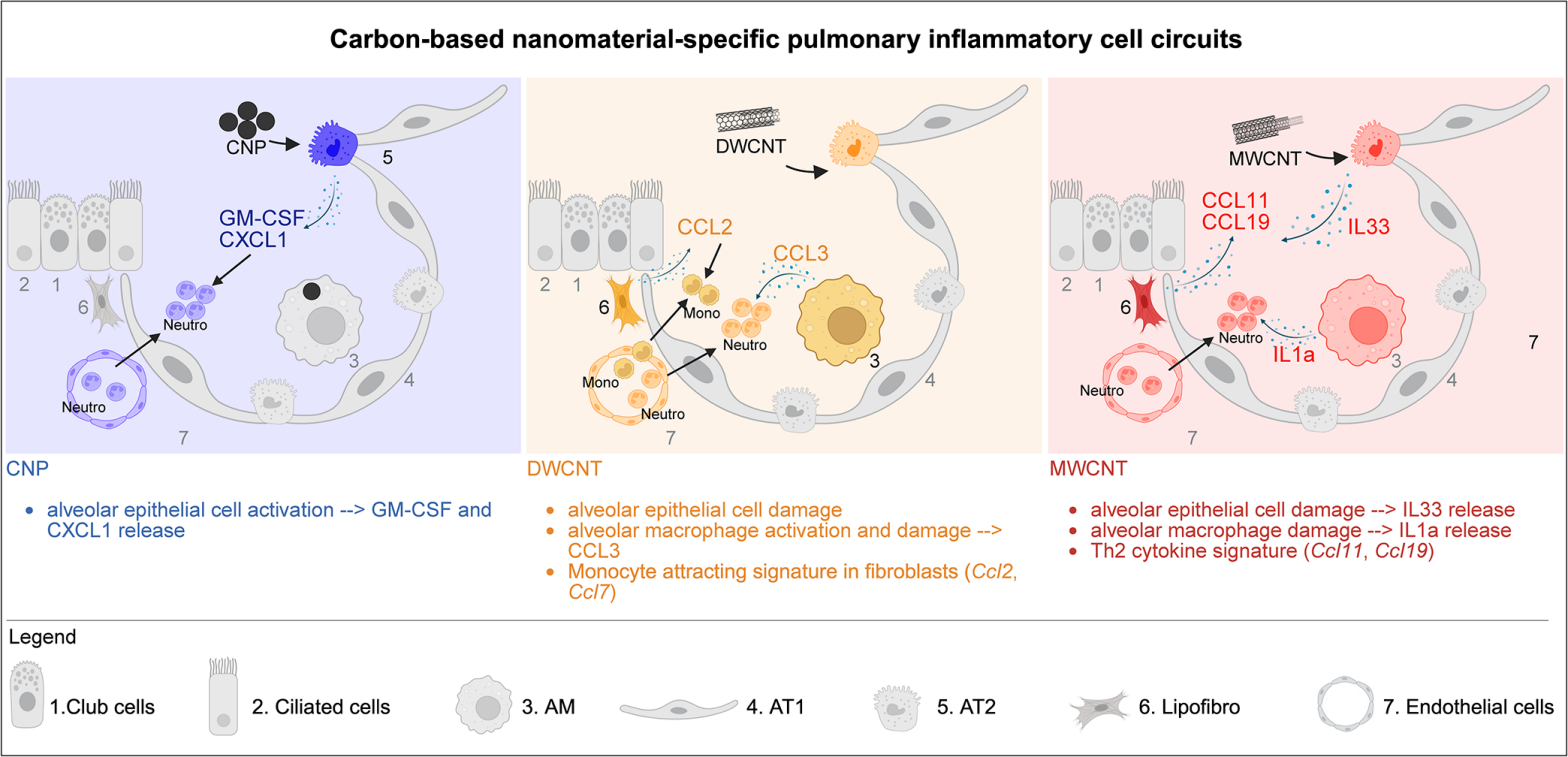
The illustration of CBN-specific pulmonary inflammatory cell circuits. The graphic illustration of CBN-specific pulmonary inflammatory responses and cell circuits.

## Materials and Methods

### Animal Study

Female C57BL/6J mice were purchased from Charles River Laboratories (Sulzfeld, Germany) at 8 weeks old and housed in individually ventilated cages according to standard operating procedures until used in this project. All animal experiments were performed following the protocols evaluated and approved by the District Government of Upper Bavaria (Ethics Approval Number: ROB-55.2–2532.Vet_02–15–67).

### Cell Culture

Murine epithelial lung tissue cell lines (LA-4; cat. no. ATCC CCL-196; MLE-12, cat no. CRL-2110), murine fibroblast cell line (CCL-206; cat. no. Mlg 2908), and murine macrophage cell line (J774.1; cat. no. TIB-67) were purchased from American Type Culture Collection (ATCC) instructions. LA-4 cells were cultured in Ham’s F-12K medium (Gibco, Darmstadt, Germany) supplemented with 15% fetal bovine serum (FBS; PAN Biotech, Aidenbach, Germany), 1% NEAA (Gibco, Darmstadt, Germany) and 100 U/mL Penicillin and 100 μg/mL Streptomycin (1% P/S). MEL12 cells were cultured in RPMI 1640 (Gibco, Darmstadt, Germany) supplemented with 10% FBS and 1% P/S. CCL-206 cells were cultured in DMEM/F12 medium (Gibco, Darmstadt, Germany) supplemented with 10% FBS and 1% P/S. J774.1 cells were cultured in DMEM (Gibco, Darmstadt, Germany) supplemented with 10% FBS, 2 mM l-Glutamine (Gibco, Darmstadt, Germany) and 1% P/S. Murine primary alveolar cells, particularly AM, recovered from BAL were cultured in RPMI 1640 medium supplemented with 10% FBS, 2 mM l-Glutamine, 0.1% β-Mercaptoethanol (2-Me; Gibco, Darmstadt, Germany), and 1% P/S. Murine primary epithelial cells (EpCAM+ cells) were cultured in the following medium: DMEM/F12 containing 100 U/mL penicillin and 100 μg/mL streptomycin, 2 mM l-alanyl-l-glutamine (Gibco), Amphotericin B (Gibco), insulin-transferrin-selenium (Gibco), 0.025 μg/mL recombinant human EGF (Sigma-Aldrich), 0.1 μg/mL Cholera toxin (Sigma-Aldrich), 30 μg/mL bovine pituitary extract (Sigma-Aldrich), and 0.01 μM freshly added all-trans retinoic acid (Sigma-Aldrich). 10 μM Y-27632 (Tocris), a Rho-associated kinase (ROCK) inhibitor, was added for the first 24 h of culture. All cells were cultured at 37 °C in a humidified environment at 5% CO_2_.

### Carbon-Based Nanomaterials (CBN)

For pulmonary CBN treatments, spherical carbon nanoparticles (CNP; Printex90, Degussa, Frankfurt, Germany), double-walled carbon nanotubes (DWCNT; Nanocyl 2100, Auvelais, Belgium), and multiwalled carbon nanotubes (MWCNT; Mitsui-7, Hodogaya Chemicals, Japan) were used. All particles were suspended in pyrogen-free water containing 0.5 mg/mL porcine lung surfactant containing all four surfactant proteins (SP; SP-A, -B-, -C, and -D). All CBN were dispersed by ultrasonic treatment as described earlier.^30^ Dispersion quality including average size (Z-Ave) and size distribution (polydispersity index = PdI) was assessed by dynamic light scattering (Zetasizer Nano ZS, Malvern Instruments Ltd., Malvern, U.K.). Detailed information on CBN physicochemical characterizations and dispersion quality was provided in Table 1.

### Mouse Lung Instillation

Seven 10–12 week old, randomly grouped mice (4 animals per DropSeq and histopathology analysis; additional 3 animals for BAL lavage analysis) were anesthetized with midazolam/medetomidine/fentanyl (MMF) and instilled intratracheally with 50 μL of either CNP (20 μg), MWCNT (15 μg), DWCNT (50 μg), 0.1 μg LPS or sham (0.5 mg/mL lung surfactant in water) per mouse as published previously.^27^ Whole lung tissue was harvested from 4 mice per group after 12 h for live single-cell and tissue analyses (Drop Seq, right lung) or fixed for histology (left lung). Bronchoalveolar lavage (BAL) fluid and cells from 3 mice per group were collected in parallel by cannulating the trachea and rinsing the lung six times with ice-cold PBS for differential BAL cell counts and protein analysis.

### Differential Cell Counts and Protein Analysis

BAL cells were pelleted by centrifugation, and supernatants were used for protein analyses as described earlier.^30^ Cells were resuspended in 1 mL of cell culture medium and counted with trypan blue (Gibco, Grand Island, NY) exclusion. Cytospins were prepared by spinning 30,000 cells per slide and stained with May-Grünwald-Giemsa staining (Merck KGaA, Darmstadt, Germany). 2*200 cells were identified by morphology and counted at 20× magnification (Olympus BX51). Total numbers of neutrophils, macrophages, multinucleated macrophages, lymphocytes, and eosinophils were assessed. Total protein content in BAL fluid after cell removal was assessed using the Pierce BCA Protein Assay Kit (Thermo Fisher Scientific, Rockford) according to the manufacturer’s instructions.

### Lung Tissue Analysis

#### Immunohistochemistry

Left lungs were filled by intratracheal instillation with 4% Paraformaldehyde (PFA; Thermo Fisher Scientific, Rockford) solution by gravitational flow, sutured and fixed overnight at 4 °C and consecutively transferred to PBS until embedding. Lung tissue was embedded in paraffin and cut into 3 μm sections. After deparaffinization and rehydration as described before,^30^ heat-induced epitope retrieval (HIER) in citrate buffer (pH = 6.0) followed, then the sections were incubated with blocking buffer (Rodent Block M; Biocare Medical/Zytomed Systems, Berlin, Germany) and labeled with primary antibody at 4 °C overnight. Sections were incubated with Rabbit-on-rodent-AP-polymer (Biocare Medical/Zytomed Systems, Berlin, Germany) after washing, followed by Vulcan Fast Red Chromogen (Biocare Medical/Zytomed Systems, Berlin, Germany). All sections were counterstained with Haematoxylin (Merck KGaA, Darmstadt, Germany). All slides were imaged using a light microscope (Olympus BX51).

#### TUNEL Assay, Immunofluorescence Double-Staining and Quantification

Left lungs were filled by intratracheal instillation with 4% Paraformaldehyde (PFA; Thermo Fisher Scientific, Rockford) solution by gravitational flow, sutured, fixed overnight at 4 °C, and consecutively transferred to PBS until embedding. Lung tissue was embedded in paraffin and cut into 3 μm sections. After deparaffinization and rehydration as described before,^30^ heat-induced epitope retrieval (HIER) in citrate buffer (pH = 6.0) followed, then the sections were incubated with blocking buffer (Rodent Block M; Biocare Medical/Zytomed Systems, Berlin, Germany). For the assessment of cell death-induced DNA fragmentation of lung cells, the terminal deoxynucleotidyl transferase dUTP nick end labeling (TUNEL) assay was performed according to the manufacturer’s instructions (ab66110, Abcam, Cambridge, Massachusetts) on paraffin-embedded lung tissue sections. Counterstaining was performed with Phalloidin (Thermo Fisher Scientific, Rockford) and DAPI (Sigma-Aldrich). For immunofluorescence (IF) double-staining with TUNEL cosignal, sections were costained with AT1 cell marker (rabbit AQP5; 1:100, Merck Millipore, 178615), AT2 cell marker (rabbit Pro-SPC, 1:200 dilution, Merck Millipore, 3786) or AM marker (rabbit CD11c, 1:200, Cell Signaling, #97585). An Olympus BX51 fluorescence microscope was used for direct visualization of dUTP-labeled DNA. Nuclei were counterstained with DAPI. TUNEL quantification was performed by taking 6 random fields of view per mouse lung. The number of TUNEL-positive cells was normalized to the DAPI events for each field. The mean of the 6 fields per lung is indicated at the individual dot in the graph.

#### Detection of CBN in Lung Tissue by Enhanced Darkfield Microscopy

The distribution of CBN in lung sections stained with TUNEL, Phalloidin, and DAPI was examined using the dual-mode fluorescence of the Cytoviva enhanced darkfield hyperspectral system (Auburn, AL). Images were acquired 40× and 100× on an amorphous Olympus BX 43 microscope with a Qimaging Retiga4000R camera. When imaging agglomerates of CNP and DWCNT, the dark-field condenser was positioned closer to the microscope slide than the standard position to enhance the ratio of CBN to tissue signal intensity.

### Measurements of Inflammatory Mediators

For cytokine and chemokine profiles in BAL fluid at 12 h after CBN treatment, we used the multiplex bead array system Bio-Plex Pro Mouse Chemokine Assay Panel 31-Plex (no. 12009159, Bio-Rad Laboratories GmbH), according to the manufacturer’s instructions. All cytokines included in the kit are shown in Table S2. Data acquisition was performed by the Luminex200 system with BioPlex Manager 6.1 software. After fitting standard curves using the logistic-5PL regression type, all data were visualized as a heatmap generated by R (v4.4.4) with the pheatmap package (v1.0.12). To assess protein concentration in BAL fluid across all time points, we used enzyme-linked immunosorbent assay (ELISA) for CXCL1, CXCL2, CXCL5, CCL2, CCL3, IL1α, IL1β, and IL-33 (R&D Systems) according to the protocols provided by the manufacturer.

### *In Vitro* CBN Treatments and Assays

#### Viability Measurement in CBN-Treated LA-4 Cells

LA-4 cells were seeded at 5000 cells/well in 96-well plates and treated with either CNP (25, 50, and 100 μg/mL), DWCNT (25, 50, and 100 μg/mL), or MWCNT (1, 2, 4, 8, 16, 32, and 64 μg/mL) dispersed in complete medium for 24 h. Cell viability was determined by using the WST-1 assay according to the manufacturer’s instructions (Roche Diagnostics, Mannheim, Germany). After the cell supernatants were removed for cytotoxicity measurements, 200 μL of WST-1 reagent diluted 1:15 in medium was added and incubated with the cells for 15 min at 37 °C. The assay mixture was centrifuged at 14,000 rpm for 10 min to remove CBN prior to measurement. Enzymatic conversion of WST-1 reagent was determined using a Microplate Reader (TECAN Group Ltd. Maennedorf, Switzerland) at 450 nm with 630 nm as the reference.

#### MLE12, CCL-206 and J774.1 Cell CBN Exposure Experiment

Cells are seeded into 12-well plates and incubated overnight at 37 °C in a humidified environment at 5% CO_2_. Supernatant was removed and cells were treated with CNP (50 μg/mL), DWCNT (50 μg/mL), and MWCNT (30 μg/mL). LPS (1 μg/mL), IL1α (10 ng/mL), and TNFα (20 ng/mL) were included as positive controls. MLE12 cells were exposed to mouse recombinant Annexin A1 (ANXA1) protein (BIOZOL; 10, 50, and 100 ng/mL) to measure *Cxcl1* expression. Cell supernatant was collected after 6 and 24 h for CXCL1 ELISA; a cell pellet was collected to isolate RNA for the qPCR experiment.

#### Murine Primary Lung Epithelial Cells Isolation and Treatment

Mice were anesthetized with 100 μL of a lethal anesthesia mixture containing ketamine (150 mg/kg) and xylazine (10 mg/kg) and were euthanized painlessly by blood withdrawal from the inferior vena cava. Blood was removed from the lungs by flushing with PBS, and the lungs were inflated intratracheally with an enzyme mix containing Dispase (Corning), Elastase (Serva), Collagenase (Sigma-Aldrich), and DNase (Applichem) followed by 1% low gelling temperature agarose (Sigma-Aldrich). The lungs were then excised and minced, and the cell suspension was filtered through nylon meshes of 100 and 40 μm. Red blood cells were removed using the RBC lysis buffer (Invitrogen). Macrophages, white blood cells, and endothelial cells were eliminated by using consecutive magnetic bead sorting with CD45 and CD31 beads (Miltenyi Biotec), respectively. Epithelial cells were enriched using CD326 (EpCAM) beads (Miltenyi Biotec) following the manufacturer’s instructions. Primary EpCAM+ cells were seeded into a 24-well plate and cultured for 24 ht at 37 °C in a humidified environment at 5% CO_2_. Supernatant was discarded and cells were treated with CNP (50 μg/mL), DWCNT (50 μg/mL), and MWCNT (30 μg/mL). After 12 h, cell pellets were collected and RNAs were isolated to measure *Csf2*, *Cxcl1* and *Il33* mRNA levels by qPCR.

#### Murine Primary Alveolar Macrophages Experiment

BAL cells of untreated mice were recovered from an untreated group of mice, as described above. Primary AMs were counted and seeded at 50.000 cells/cm^2^ in 24-well plates. After 3 h, attached cells were washed twice with warm cell culture medium and treated with CBN preparations diluted in the cell culture medium for 24 h. Supernatants were collected, centrifuged to remove CBN suspended in the medium, and used for Il1α ELISA measurements as described above.

#### cDNA Synthesis and qPCR

Whole cell RNA was isolated with a NucleoSpin RNA Plus kit (MACHEREY-NAGEL, Duren, Germany) following the instructions of the manufacturer. RNA was quantified using a Nanodrop (Thermo Fisher Scientific, Waltham, MA). Next, cDNA synthesis was performed using a superscript kit (Invitrogen, Waltham, MA) and subsequently used to analyze the target gene expression by real-time quantitative PCR (qPCR) using SYBR Green PCR master mix (Thermo Fisher Scientific, Waltham, MA). Primer pairs used for qPCR are shown in Table S1.

### RNA Sequencing of CBN-Treated Lungs at Single-Cell Resolution

#### Generation of Single-Cell Suspensions

Lung single-cell suspensions for Dropseq were generated from right lung lobes as previously described.^16^ Briefly, the right lung lobes were removed and minced before undergoing enzymatic digestion in a mix of Dispase, collagenase, elastase, and DNase for 20–30 min at 37 °C with agitation. Following straining of the cell suspension through a 40 μm mesh filter, cells were centrifuged and counted in PBS with 10% FCS. For Dropseq, cell aliquots in PBS supplemented with 0.04% bovine serum albumin were prepared with a cell density of 100 cells/μL.

#### Single-Cell RNA-Sequencing by Dropseq

Dropseq experiments followed the original protocols, using adaptations and the microfluidic device as described in detail earlier.^15^ Quality controls, including the number of unique molecular identifiers (UMI), as well as detection of genes per cell and reads that can be aligned to the mouse genome, were met for all mice. Every treatment was analyzed together with control mice that were instilled with sham controls (lung surfactant in water). Counting of mRNA copies with UMI was performed to determine the differential gene expression between single cells. For processing of the whole-lung data set, the computational pipeline of Dropseq was used as described before (version 2.0).^17^

### Analysis of the Whole-Lung Data Set

All the analyses were performed using Scanpy (version 1.8.0) and relative complementary tools. Martrice of each sample was concentrated followed by quality control (QC). All of the sham groups were pooled together. During QC, genes with fewer than 1 count and were expressed in less than 5 cells were removed. Next, cells with greater than or equal to 10% mitochondrial counts, fewer than 300 genes, or fewer than 500 total counts were removed. Did we do doublet detection with Scrublet. Then the log transformation was performed using the Scanpy pp.log1p­() function. High-variable genes (HVG) were computed for each sample and were considered as overall HVGs only if they were expressed at least in two samples. HVGs were then used for Principal Composition Analysis (PCA) to create a kNN graph with the first 50 principal components. Then a graph-based clustering using the Leiden algorithm was performed,^52^ clusters were annotated with classical marker genes. Briefly, the whole lung data were annotated to four different subsets: epithelial cells (*Epcam*+), endothelial cells (*Cldn5*+), stromal cells (*Col1a*2+), and immune cells (*Ptprc*+). Then, for fine cell type annotation, a subsequent repetition of HVG selection, PCA and graph-based clustering were performed to achieve fine annotations of each subset. Marker genes were computed using a Wilcoxon rank-sum test, and genes were considered marker genes if the FDR-corrected p-value was below 0.05 and the log2 fold change (log2FC) was above 0.5. The top 500 gene list of each annotated cell type is shown in Supporting Table S3.

### Analysis of Differentially Expressed Genes

Differential expression analysis was performed with diffxpy (v.0.7.4). Welch’s *t* test with default parameters was used to compare expression differences between two groups for all genes that were expressed in at least 10 cells. Differentially expressed genes are labeled if the FDR-corrected *p*-value is less than 0.05. Genes with log2FC of more than 0.5 or less than *–*0.5 were considered for further analysis.

### Gene Set Variation Analysis

Gene Set Variation Analysis was performed to estimate the variation of gene set enrichment of hallmark signaling pathways either in the whole lung or specific cell niche/state. Gene Set Variation Analysis results were visualized by a Uniform Manifold Approximation and Projection.

### Gene Set Enrichment Analysis

After Differential gene expression (DGE) analysis, gene log2FC and FDR-corrected p values from each cell type were collected for Gene Set Enrichment Analysis. It was performed with the clusterProfiler package (version 4.0)^53^ using the following libraries from the mouse database:’MSigDB_Hallmark_2022’,’GO_Biological_Process_2021’. Gene sets were considered enriched in the respective signature if the FDR-corrected p-value was less than 0.05.

### Gene Signaling Scoring Analysis

The score of the gene signaling signature is calculated by an average of a certain set of genes, using Scanpy’s sc.tl.score_genes­() function with default parameters. This analysis was applied for damage associated molecular pattern (DAMP) score, genes related to DAMP score were listed in Table S4. Scoring of signaling from public databases of “MSigDB_Hallmark_Inflammatory response (MM3890)”, “GO_Inflammatory response (GO:0006954)”, and ″MSigDB_Hallmark_TNFA_SIGNALING_VIA_NFKB (MM3860)” were used in the study.

### Cell-Cell Communication by Induced Connectome Analysis

To identify cell–cell communication networks, a list of annotated receptor–ligand pairs was downloaded, which was listed in Supporting Table S5. Next, we integrated this information with the cell type differentially upregulated genes with log2FC values greater than 0.5. Cell-cell communication networks were generated in the following manner. An edge was created between two cell types if these two cell types shared a receptor–ligand pair as differentially upregulated genes.

### Regulatory Potential and NicheNet Analysis

NicheNet analysis on the scRNA seq data using the R (version 4.2.2) packages nichenetr (version 1.0), according to the literature.^54^ Based on DEG analysis, differentially expressed genes with log2FC more than 0.5 and FDR-corrected *p*-value <0.05. For each receiver cell type, the respective perturbed gene programs were then used as input for NicheNet to predict ligands that could induce the gene program in the receiver cell type. We only considered ligands expressed by at least 10% of sender cells and had a Pearson correlation prediction ability >0.05. Significantly expressed ligands were selected, and the ligand–receptor pairs between different cell types were visualized by the R circlize package (v0.4.15).

### Webtool

The webtool was created using the R package ShinyCell (version 2.1.0) and the R package schard (version 0.0.1) for converting the scRNASeq data format.

### Softwares

R (v4.2.2), Adobe Illustrator 2022, ImageJ, Zen (3.3), and GraphPad Prism (v9.1.0) were used for statistical analysis and imaging visualization.

### Statistical Analysis

For *in vivo* pathological analysis, data are shown as mean ± SEM. Comparisons between two groups were performed using Student’s *t* test. Comparisons between multiplex groups were performed using One-way ANOVA (nonparametric analysis; Kruskal–Wallis Test) followed by Dunn’s multiple comparisons test were used for statistical analysis. Statistical analysis was performed using GraphPad Prism software v10.1.2 (GraphPad Software, Inc., San Diego, CA). *P* value was shown and *P* < 0.05 was considered a significant statistical difference.

## Supplementary Material









## Data Availability

All scRNA-seq raw data have been submitted to GEO (GSE273313). All other data supporting the findings of this study are available within the Article and Supporting Information. The CBN-specific modes of action described in our work are accessible through our published webtool (https://organoidtox.shinyapps.io/nanoparticle_only_exposure_app/). All codes used for scRNA-seq data visualization have been uploaded to GitHub and are freely accessed via: https://github.com/LianyongHan/Carbon-Based-Nanomaterials-and-the-Initiating-Cell-Circuits-in-Pulmonary-Inflammation.

## Notes

Activation of pro-inflammatory response in different lung cell niches (Figure S1); CBN-specific activation of cytokine responses (Figure S2); pro-inflammatory responses in epithelial niche caused by CBN (Figure S3); CNTs caused epithelial niche damage (Figure S4); pro-inflammatory activation in mesenchymal niche caused by CBN (Figure S5) (PDF)
ToxAtlas webtool tutorial; Webtool tutorial to explore CBNNM-specific gene expression in single-cell transcriptomic data (PDF)
Top 500 gene list of each annotated cell type (Table S3) (XLSX)
Ligand–receptor pairs for cell–cell communication analysis (Table S5) (XLSX)
