# Supplementary material for: Toward a ToxAtlas of Carbon-Based Nanomaterials: Single-Cell RNA Sequencing Reveals Initiating Cell Circuits in Pulmonary Inflammation
Source: ACS Nano. 2025 Nov 3;19(45):39139–56. doi: 10.1021/acsnano.5c12054 (PMC12632174; doi:10.1021/acsnano.5c12054)
Supplement: Supplementary file 1 [file nn5c12054_si_001.pdf]

Toward a ToxAtlas of Carbon-Based Nanomaterials: Single-Cell RNA Sequencing Reveals Initiating Cell Circuits in Pulmonary Inflammation

Carola Voss[1,2,3]*, Lianyong Han[1]*, Meshal Ansari[1,4,12], Maximilian Strunz[1,12], Verena Haefner[1], Ilias Angelidis[1,12], Christoph H. Mayr[1,12], Trine Berthing[5], Qiaoxia Zhou[1], Eva M. Guenther[1], Osama Huzain[1], Otmar Schmid[1], Ulla Vogel[5], Janine Gote-Schniering[1,7,12], Svenja Gaedcke[3,8], Fabian J. Theis[4,9,10,11], Herbert B. Schiller[6,12#] and Tobias Stoeger[1#]

[1]Institute of Lung Health and Immunity (LHI), Comprehensive Pneumology Center (CPC), Helmholtz Center Munich, Neuherberg 85764, Germany; German Center for Lung Research (DZL), Munich 81377, Germany

[2]Hannover Medical School, Clinic for Cardiac, Thoracic, Transplantation and Vascular Surgery, Leibniz Research Laboratories for Biotechnology and Artificial Organs (LEBAO), Hannover 30625, Germany

[3]Biomedical Research in Endstage and Obstructive Lung Disease Hannover (BREATH), German Center for Lung Research (DZL), Hannover 30625, Germany

[4]Department of Computational Health, Institute of Computational Biology, Helmholtz Center Munich, Neuherberg 85764, Germany

[5]National Research Centre for the Working Environment, Copenhagen 2100, Denmark

[6]Institute of Experimental Pneumology, LMU University Hospital, Ludwig-Maximilians University, Munich 81377, Germany

[7]Department of Rheumatology and Immunology, Department of Pulmonary Medicine, Allergology and Clinical Immunology, Inselspital, Bern University Hospital, University of Bern, Bern 3012, Switzerland; Lung Precision Medicine, Department for BioMedical Research, University of Bern, Bern 3012, Switzerland

[8]Department of Respiratory Medicine and Infectious Diseases, Hannover Medical School; Hannover 30625, Germany

[9]School of Life Sciences Weihenstephan, Technical University of Munich, Munich 35354, Germany

[10]School of Computing, Information and Technology, Technical University of Munich, Munich 35354, Germany

[11]Wellcome Sanger Institute, Wellcome Genome Campus, Cambridge CB10 1SA, UK

[12]Research Unit for Precision Regenerative Medicine, Helmholtz Center Munich, Munich 81377, Germany; Comprehensive Pneumology Center Munich (CPC), German Center for Lung Research (DZL), Munich 81377, Germany

*equal contribution

#Corresponding authors: herbert.schiller@helmholtz-munich.de and tobias.stoeger@helmholtz-munich.de

Supplementary Figures

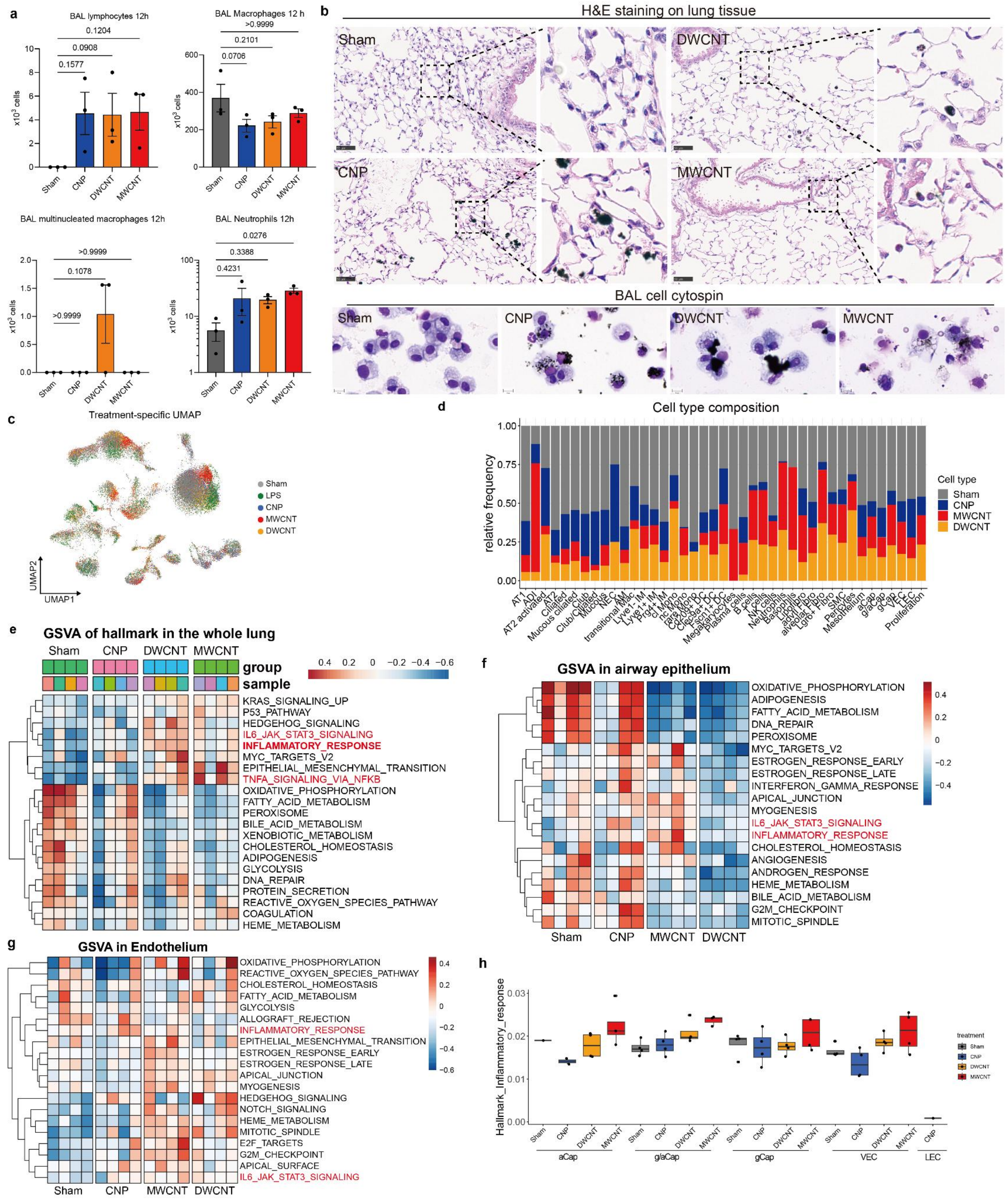

Figure S1 The activation of pro-inflammatory response in different lung cell niches

a. Differential cell counts in BAL of CBN treated mice after 12h. **b.** CBN deposition and distribution in the lung tissue and BAL cells visualized by lung H&E staining and BAL cell cytospin staining, respectively. **c.** Visualization of treatment-specific cellular response patterns by UMAP. **d.** The composition of 41 cell types in the mouse lung shown in a treatment-specific manner. **e.** Gene set variation analysis (GSVA) with the whole lung on signaling pathway in hallmark database. **f.** GSVA analysis of signaling pathways in the hallmark dataset in airway epithelium. **g.** GSVA analysis of signaling pathways in the hallmark dataset in endothelium. **h.** Scoring of "Hallmark_Inflammatory

response” in endothelial cell types (aCap, g/aCap, gCap, VEC and LEC). Data are shown as the mean ± SEM of three mice (n=3 for BAL analysis), one-way ANOVA (non-parametric analysis; Kruskal-Wallis-Test) followed by Dunn's multiple comparisons test was used for statistical analysis.

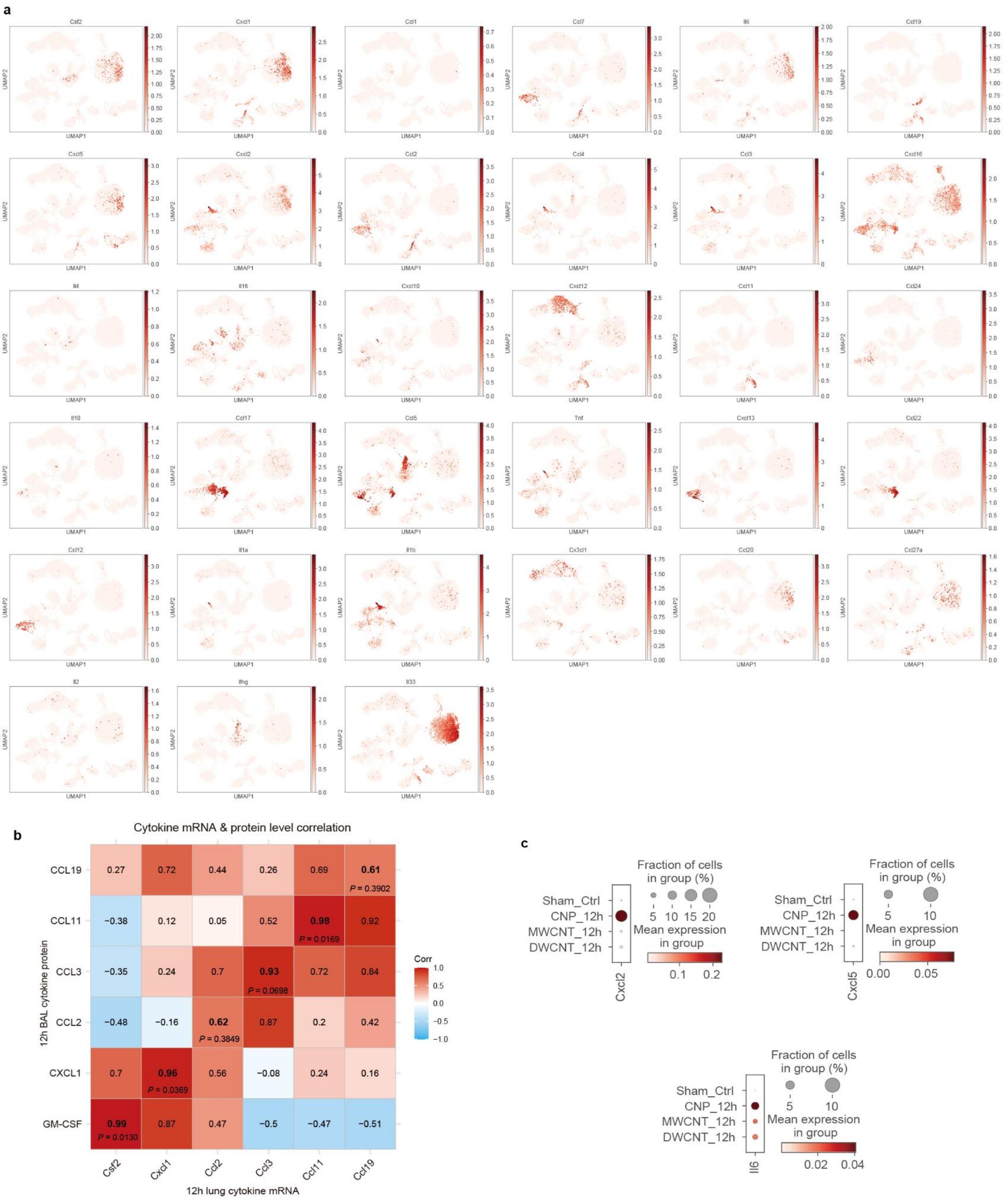

Figure S2 CBN-specific activation of cytokine responses

a. The localization of cytokine gene expression visualized by UMAP. **b.** The correlation of released BAL cytokine protein (GM-CSF, CXCL1, CCL2, CCL3, CC11 and CCL19) to RNA level (*Csf2, Cxcl1, Ccl2, Ccl3, Ccl11 and Ccl19*) in inflammatory niche. The correlation score and *P* value was shown. **c.** The induction of *Cxcl2*, *Cxcl5* and *Il6* in AT2 activated cells.

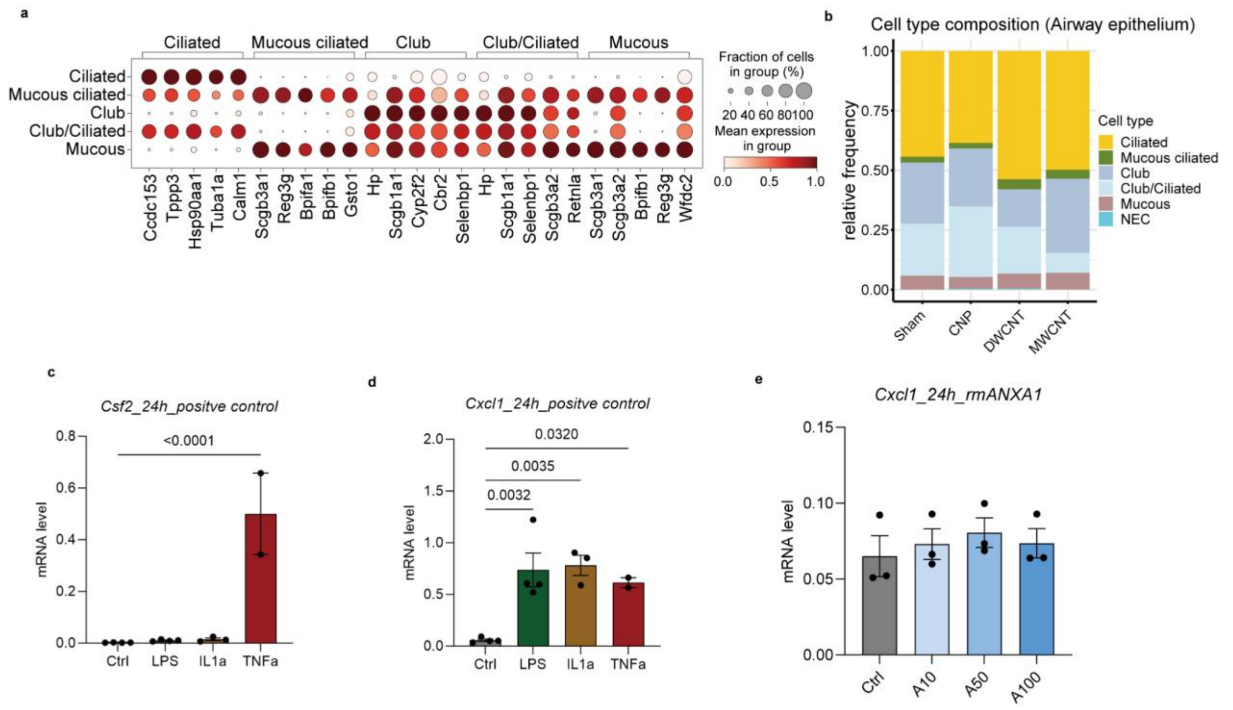

Figure S3 The pro-inflammatory responses in epithelial niche caused by CBN

a. Dotplots displaying the top 5 marker genes for each annotated cell type in airway epithelium. **b.** Relative cell frequency shows treatment-dependent changes in airway epithelium. **c.** *Csf2* induction by different positive controls including LPS (1 μg/ml), IL1a (10 ng/ml) and TNF-a (20 ng/ml) in AT2 like MLE-12 cells. **d.** *Cxcl1* induction by different positive controls including LPS (1 μg/ml), IL1a (10 ng/ml) and TNF-a (20 ng/ml) in AT2-like MLE-12 cells. **e.** *Cxcl1* induction by different concentrations of mouse recombinant ANXA1 protein (A10: 10 ng/ml, A50: 50 ng/ml, A100: 100 ng/ml). All data are shown as the mean ± SEM (n = 3 for *in vitro* experiment as well as BAL samples), one-way ANOVA followed by Dunn's multiple comparisons test was used for statistical analysis. *P* value was shown and *P* value < 0.05 was considered statistically significant.

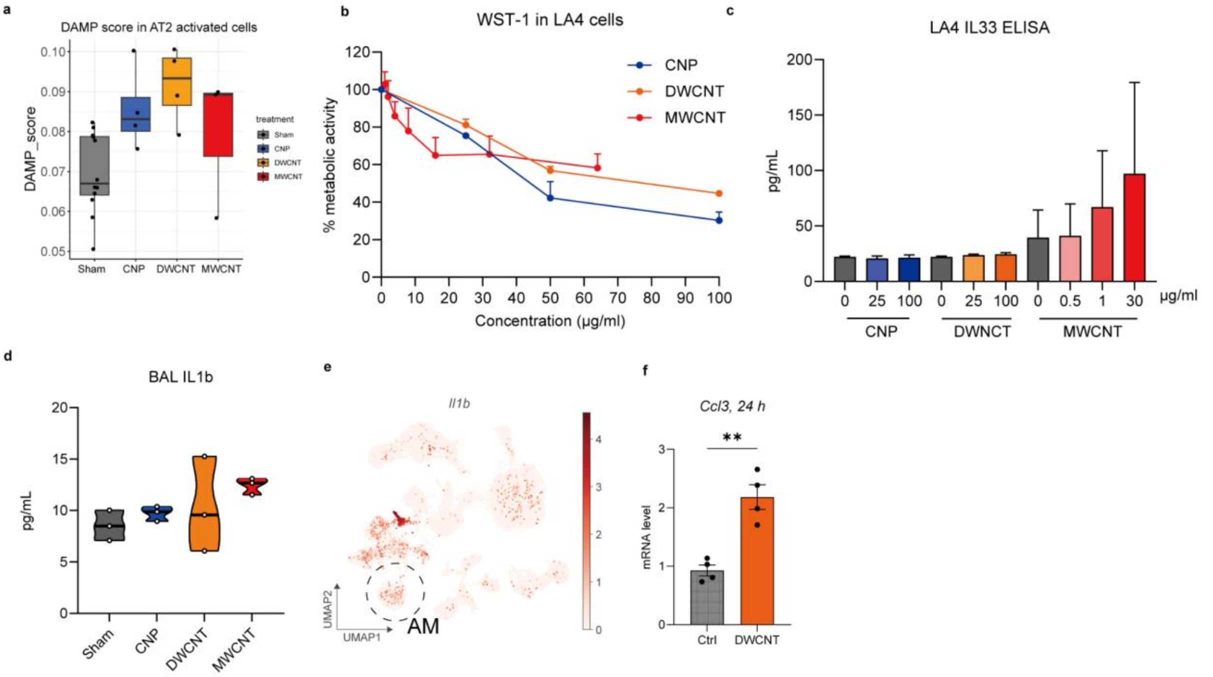

Figure S4 CNTs caused epithelial niche damage

a. DAMP score in actAT2 (AT2 activated) cells. **b.** LA-4 cell viability changes caused by different CBN measured by WST-1 assay. **c.** The release of IL33 into the supernatant of LA-4 cells caused by CBN after 24h. CNP (25, 100 µg/ml), DWCNT (25, 100 µg/ml), MWCNT (0.5, 1, 30 µg/ml). **d.** IL1b release caused by CBN into BAL fluid measured by ELISA. **e.** The visualization of *Il1b* expression and localization. **f.** *Ccl3* induction by DWCNT in J774.1 macrophages. Data is shown as mean ± SEM (n = 3). A Student *t* test was performed between DWCNT and control group. *P* value was shown and *P* value < 0.05 was considered statistically significant.

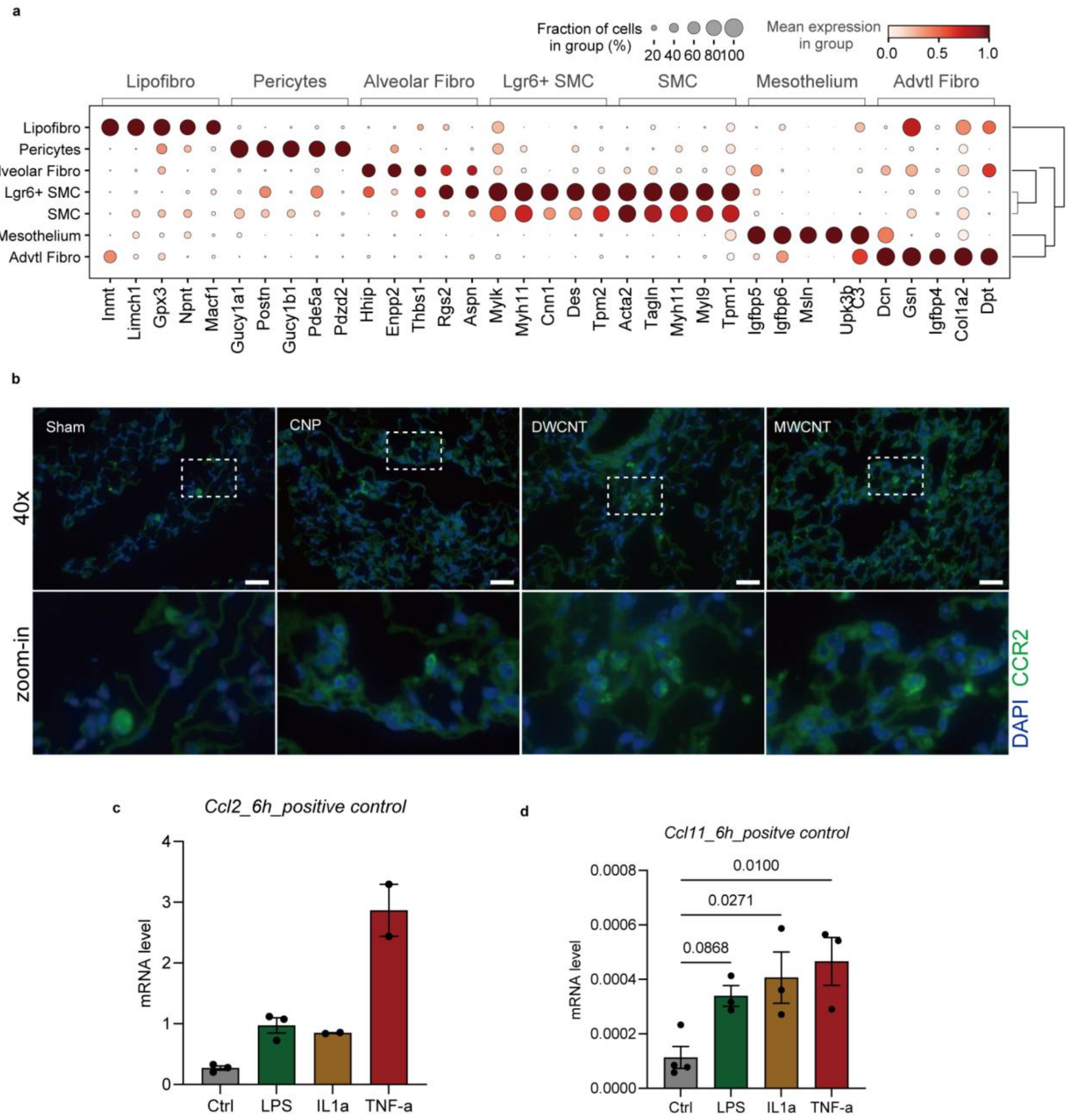

Figure S5 The pro-inflammatory activation in mesenchymal niche caused by CBN

a. Dotplots displaying the top 5 marker genes for each annotated cell type in mesenchymal cells. **b.** Immunofluorescence staining of monocyte attraction and accumulation (CCR2 positive) in the lung interstitium caused by DWCNT. DAPI: blue, CCR2: green. Scale bar: 20 µm. **c.** *Ccl2* induction by LPS (1 µg/ml), IL1a (10 ng/ml) and TNF-a (20 ng/ml) in CCL206 fibroblasts. **d.** *Ccl11* induction by LPS (1 µg/ml), IL1a (10 ng/ml) and TNF-a (20 ng/ml) in CCL206 fibroblasts. All data are shown as the mean ± SEM (n = 3 for *in vitro* experiment), one-way ANOVA followed by Dunn's multiple comparisons test was used for statistical analysis. *P* value was shown and *P* value < 0.05 was considered statistically significant.

Supplementary table

Table S1 Primer pairs used in this study

Gene name	Forward primer (5'-3')	Reverse primer (5'-3')
Cxcl1	GCTGGGATTCACCTCAAGAA	TCTCCGTTACTTGGGGACAC
Csf2	GCCATCAAAGAAGCCCTG	GCGGGTCTGCACACATGTTA
Ccl2	TTAAAAACCTGGATCGGAACCAA	GCATTAGCTTCAGATTTACGGGT
Ccl3	ACTGCCTGCTGCTTCTCCTACA	ATGACACCTGGCTGGGAGCAAA
Ccl11	GCACCCTGAAAGCCATAGTCT	TGGGGTCAGCACAGATCTCT
Ccl19	GGGGTGCTAATGATGCGGAA	CCTTAGTGTGGTGAACACAACA
Il33	TCCTTGCTTGGCAGTATCCA	TGCTCAATGTGTCAACAGACG
Hprt	GTTGGATACAGGCCAGACTTTGT	CACAGGACTAGAACACCTGC

Table S2 The Bio-Plex Pro Mouse Chemokine Panel 31-Plex

BCA-1 / CXCL13	IL-4	MIP-1α / CCL3
CTACK / CCL27	IL-6	MIP-1β / CCL4
ENA-78 / CXCL5	IL-10	MIP-3α / CCL20
Eotaxin / CCL11	IL-16	RANTES / CCL5
Eotaxin-2 / CCL24	IP-10 / CXCL10	MIP-3β / CCL19
Fractalkine / CX3CL1	I-TAC / CXCL11	SCYB16 / CXCL16
GM-CSF	KC / CXCL1	SDF-1α / CXCL12
I-309 / CCL1	MCP-1 / CCL2	TARC / CCL17
IFN-γ	MCP-3 / CCL7	TNF-α
IL-1β	MCP-5 / CCL12	
IL-2	MDC / CCL22	

Table S3 Top 500 gene list of each annotated cell type

The table is attached in a separated file

Table S4 Damage associated molecular pattern (DAMP) score gene list

Bgn	*Gpc1*	*Hmgb1*	*S100a1*	*Sap130*
Calr	*Gpc2*	*Hmgn1*	*S100a10*	*Sdc1*
Calr3	*Gpc3*	*Hspd1*	*S100a11*	*Sdc2*
Camp	*Gpc4*	*Hspd2*	*S100a13*	*Sdc3*
Dcn	*Gpc5*	*Il1a*	*S100a2*	*Sdc4*
Fga	*Gpc6*	*Il33*	*S100a3*	*Tnc*
		Lynx1	*S100a4*	*Vcan*

		Ros1	*S100a5*	
			S100a6	
			S100a8	
			S100a9	
			S100b	

Table S5 Ligand-receptor pairs for cell-cell communication analysis

The table is attached as a separated file

Abbreviations

Adverse outcome pathways (AOP)

American Type Culture Collection (ATCC)

Bronchoalveolar lavage (BAL)

Carbon-based nanomaterials (CBN)

Carbon nanoparticles (CNP)

Carbon nanotubes (CNT)

Damage associated molecular pattern (DAMP)

Differential gene expression (DGE)

Double-walled carbon nanotubes (DWCNT)

Enzyme-linked immunosorbent assay (ELISA)

Fetal bovine serum (FBS)

Heat-induced epitope retrieval (HIER)

Hematoxylin-eosin (H&E)

High-variable genes (HVG)

Immunofluorescence (IF)

Key event (KE)

Lipopolysaccharide (LPS)

Midazolam/ Medetomidine/ Fentanyl (MMF)

Molecular initiating event (MIE)

Multi-walled carbon nanotubes (DWCNT)

Mode of action (MoA)

Non-Essential Amino Acids (NEAA)

Paraformaldehyde (PFA)

Polydispersity index (PdI)

Principal Composition Analysis (PCA)

Quality control (QC)
Standard error of the mean (SEM)
Terminal Deoxynucleotidyl Transferase-Mediated dUTP Nick End Labeling (TUNEL)
Uniform Manifold Approximation and Projection (UMAP)
Unique molecular identifiers (UMI)

ToxAtlas webtool tutorial

1. Title

Gene expression mapping of carbon-based nanomaterial-specific response patterns in acute lung inflammation

2. Motivation and overview

This tutorial guides you through the use of a **web-based ToxAtlas**, an interactive application for exploring and visualizing gene expression of carbon-based nanomaterial (CBN)-specific response patterns during acute lung inflammation in our single-cell RNA sequencing data. This is a brief overview on how to navigate the interface, generate plots, and interpret results.

3. Getting started

- A browser (Chrome/Firefox/Safari etc.)
- Access the ToxAtlas *via*:

  https://organoidtox.shinyapps.io/nanoparticle_only_exposure_app/

Note: The ToxAtlas is ready to use without registration or login. This webpage is made using ShinyCell.

What you see on the main page:

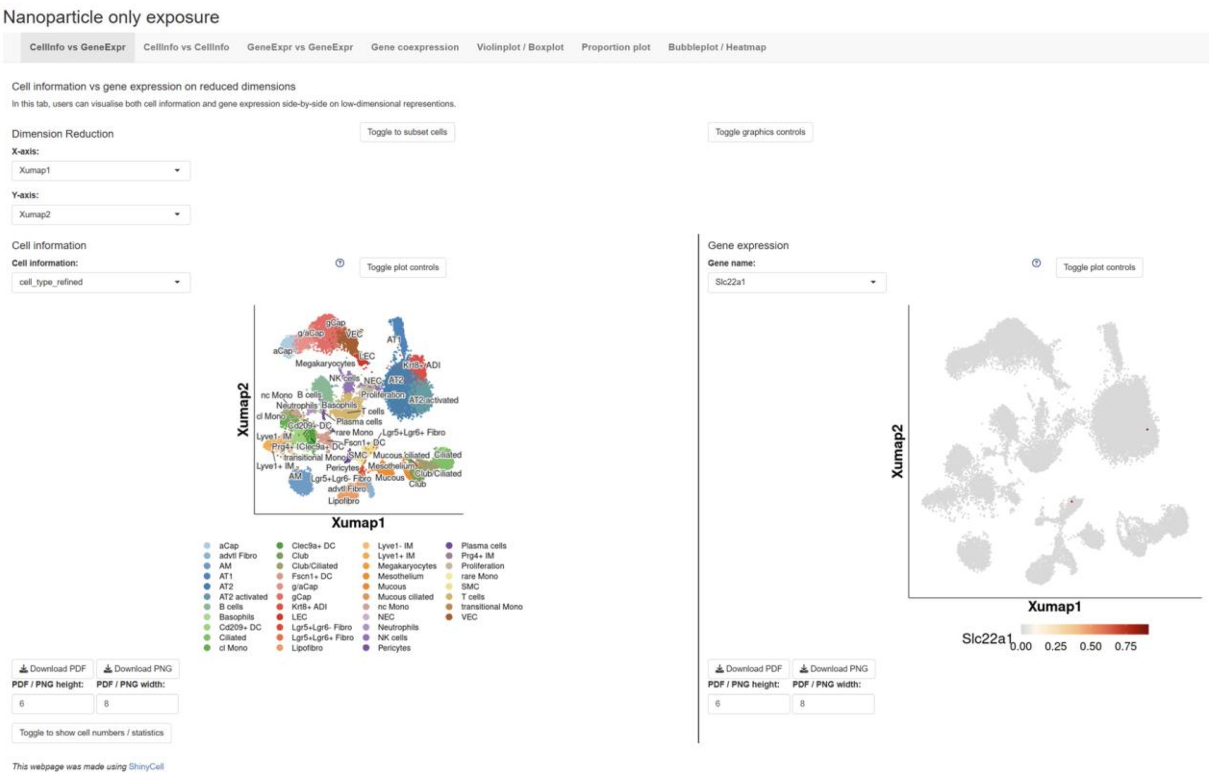

4. Interface Overview

There are seven tabs enabling you to investigate gene expression *via* different comparisons:

- "CellInfor vs GeneExpr": Cell information vs gene expression on reduced dimensions. In this tab, users can visualise both cell information and gene expression side-by-side on low-dimensional representations.
- "CellInfor vs CellInfor": Cell information vs cell information on dimension reduction. In this tab, users can visualise and compare cell information side-by-side on low-dimensional representations.
- "GeneExpr vs GeneExpr": Gene expression vs gene expression on dimension reduction. In this tab, users can visualise gene expression side-by-side on low-dimensional representations.
- "Gene coexpression": Coexpression of two genes on reduced dimensions. In this tab, users can visualise the coexpression of two genes on low-dimensional representations.
- "Violinplot / Boxplot": Cell information / gene expression violin plot / box plot. In this tab, users can visualise the gene expression or continuous cell information (*e.g.* Number of UMIs / module score) across groups of cells (*e.g.* library / clusters).
- "Proportion plot": Proportion / cell numbers across different types of cell information. In this tab, users can visualise the composition of single cells based on one discrete cell information across another discrete cell information. Usage examples include the library or cellcycle composition across clusters.
- "Bubbleplot / Heatmap": Gene expression bubbleplot / heatmap. In this tab, users can visualise the gene expression patterns of multiple genes grouped by categorical cell information (*e.g.* library / cluster). The normalised expressions are averaged, log-transformed and then plotted.

5. Performing Basic Tasks

- Visualizing clusters or cell types
- Exploring gene expression and co-expression
- Exploring treatment effect
- Exploring cell compositions

6. Example Snippet:

Generating a UMAP Plot of Cxcl1 gene expression

(1) Go to the "CellInfor vs GeneExpr" tab.

(2) Keep dimension reduction with "Xumap1" and "Xumap2", respectively.

(3) In "Cell Information", select your desired function: "cell_type_refined", "timepoint", "meta_celltype", "treat_time", "Xumap1" and "Xumap2".

(4) Go to the "Gene expression" option on the right side of the page, type in "Cxcl1" and select the correct gene.

(5) A UMAP is displayed (as below).

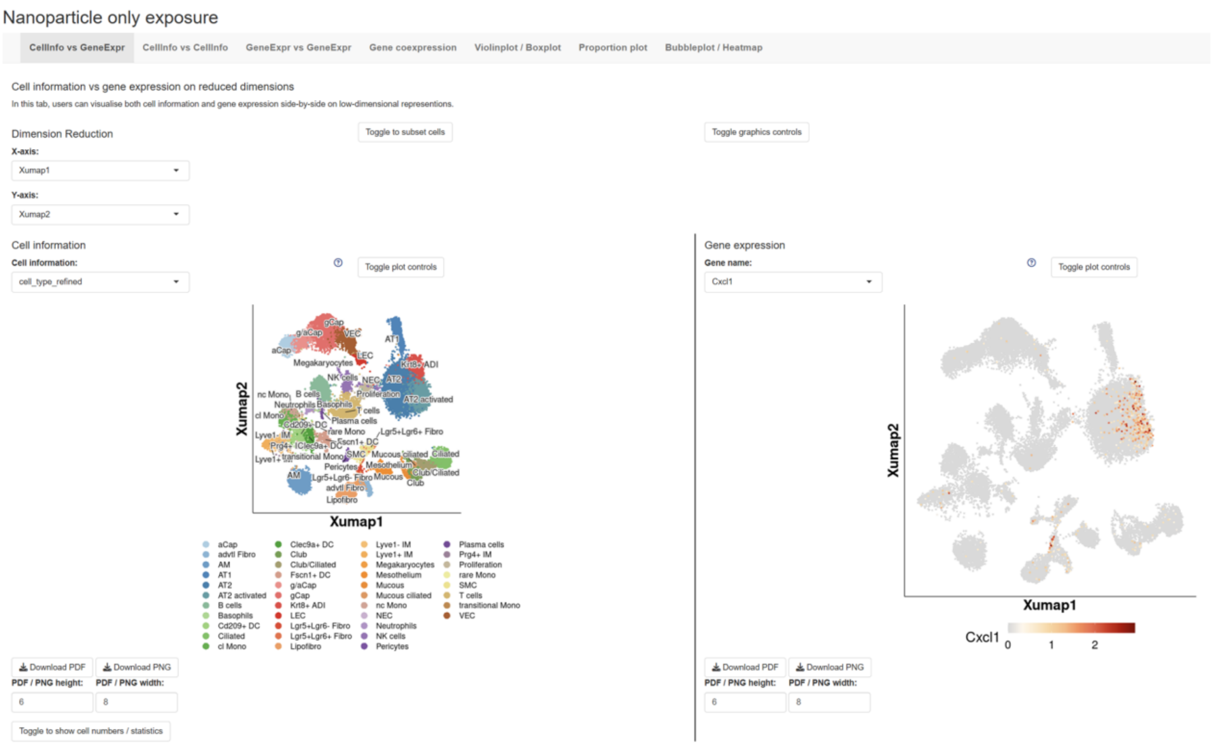

Generating a UMAP Plot of Csf2 and Cxcl1 gene coexpression

(1) Go to the "Gene coexpression" tab.

(2) Keep dimension reduction with "Xumap1" and "Xumap2", respectively.

(3) Go to the "Gene expression" option on the right side of the page, type in "Csf2" and "Cxcl1" and select the correct gene.

(4) A UMAP is displayed (as below), here the co-expression of *Csf2* (blue) and *Cxcl1* (red) are shown.

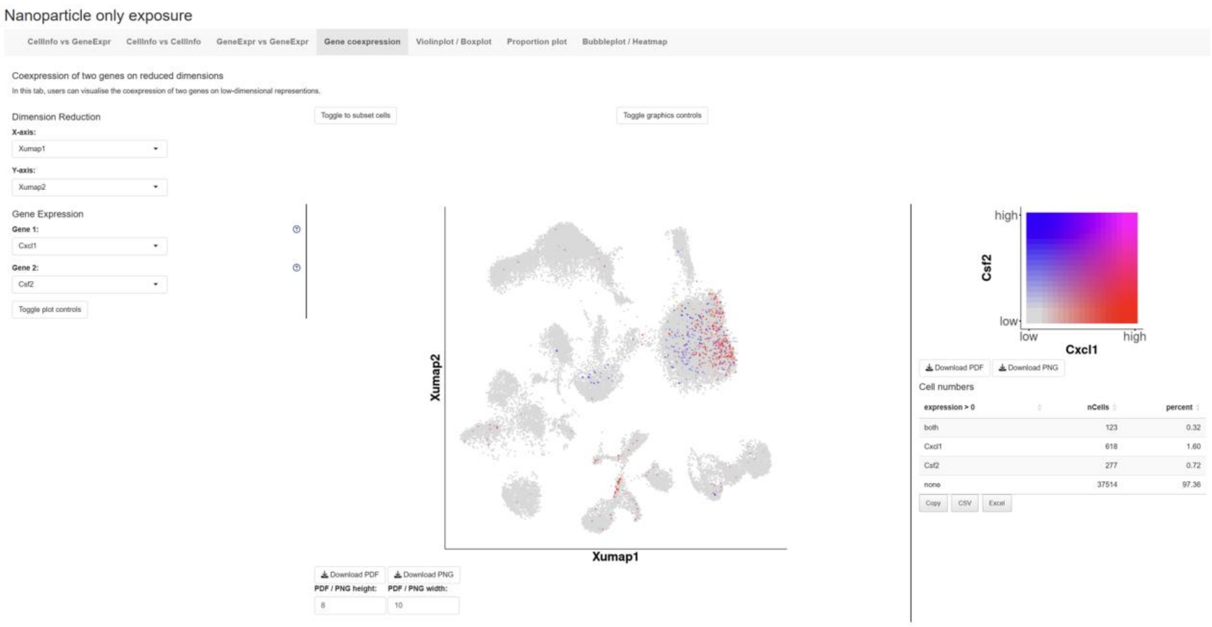

Generate a Bubbleplot to display gene expression between treatments

Here, we show the example of two specific cytokine genes for each CBN (CNP: *Cxcl1*, *Csf2*; DWCNT: *Ccl2*, *Ccl3*; MWCNT: *Ccl11*, *Ccl19*)

(1) Go to the "Bubbleplot/Heatmap" tab;

(2) Find the box under "List of gene names (Max 50 genes, separated by , or ; or newline):" and type in Cxcl1, Csf2, Ccl2, Ccl3, Ccl11, Ccl19;

(3) Select "Group by" as "treat_time" or what categories as you wish to visualize the data;

(4) Select "Plot type" as "Bubbleplot" or "Heatmap" as you wish to visualize the data;

(5) We recommend using "Scale gene expression", you can also decide to select "Cluster rows (genes)" and "Cluster columns (samples)" as you need;

(6) A bubbleplot is displayed (below).

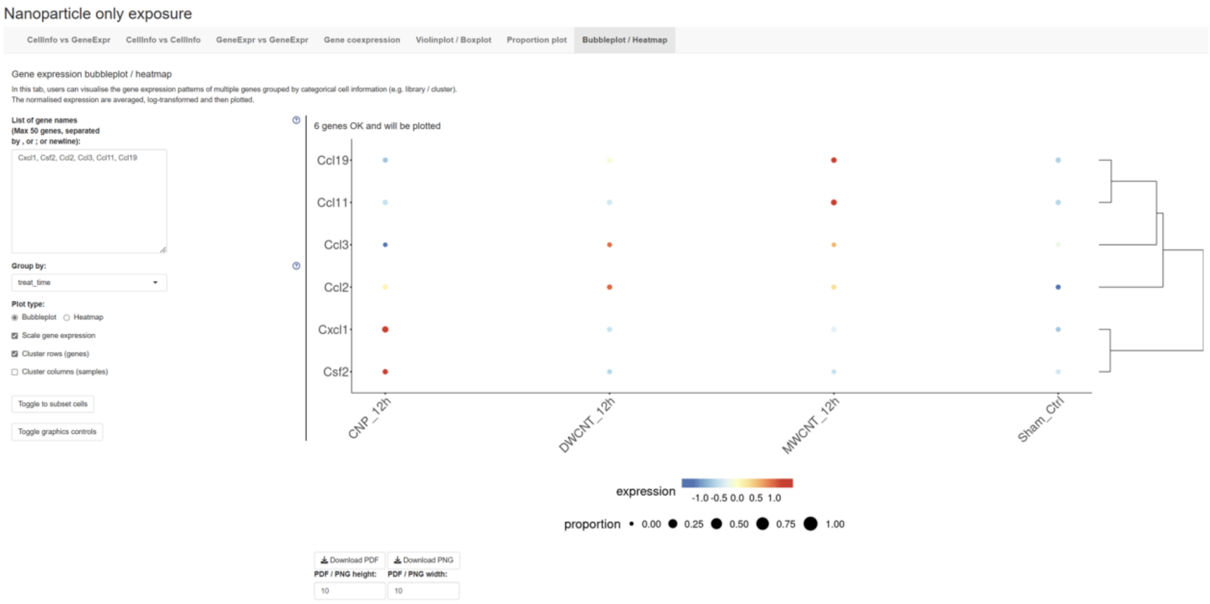

**Suggestions*

(1) Click "Toggle to subset cells", users can set a subset of any cell information included in the tool. As shown below, users can select which cells they want to be displayed on the UMAP.

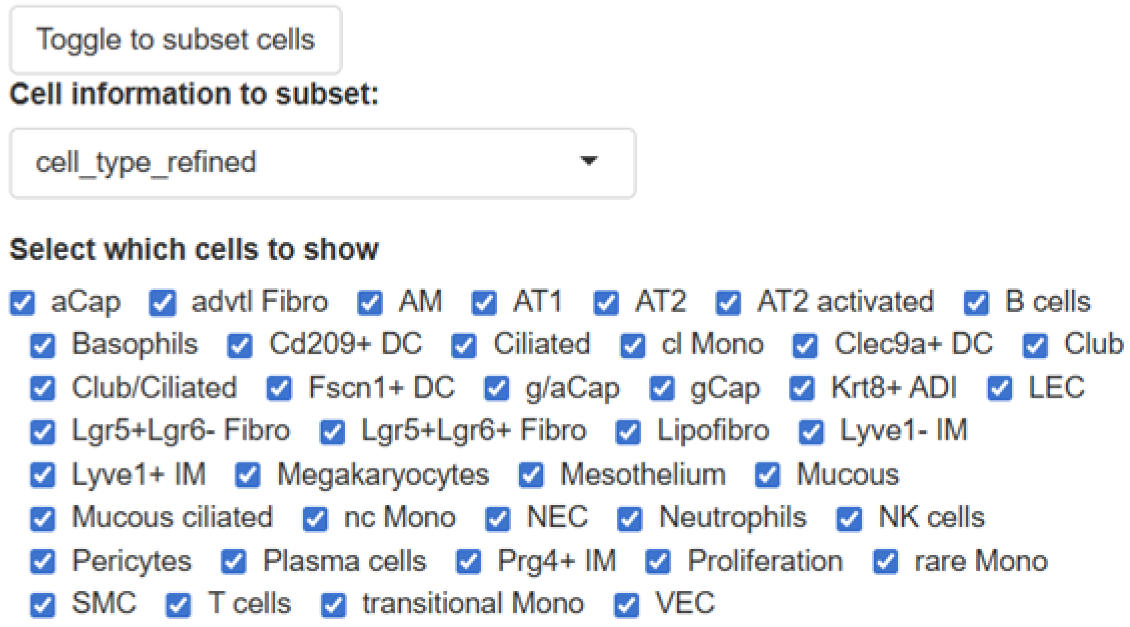

(2) The default colour palette is "White-Red", users are able to change to the other two options by clicking "Toggle plot controls". Besides, showing the highly expressed cells / dots on top of the rest on the UMAP, please set Plot order to "Max-1st" (the default setting).

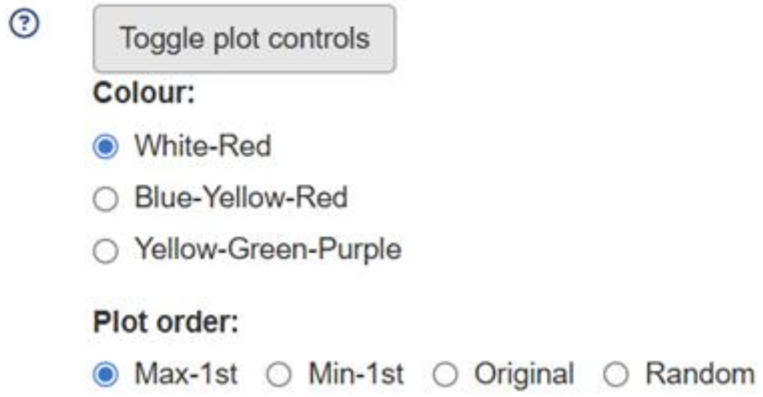

7. Interpreting Results

The UMAP on the left shows the 41 identified cell types ("cell_type_refined") annotated in the project; the UMAP on the right shows the localization of Cxcl to Krt8+ ADI and AT2 activated cells.

8. Saving and Exporting

It is possible to save plots by clicking "Download PDF" or "Download PNG" under the UMAP. Users are able to set the height and width of the exported file.

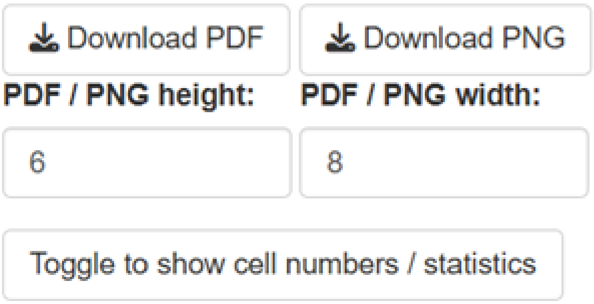

9. Resources

For feedback please contact: Dr. Tobias Stoeger tobias.stoeger@helmholtz-munich.de

Institute of Lung Health and Immunity (LHI) / Comprehensive Pneumology Center (CPC), Helmholtz Munich, Germany

Important: Please cite this publication when using exported plots from our ToxAtlas webtool for publications
