# Supplementary material for: Toward a ToxAtlas of Carbon-Based Nanomaterials: Single-Cell RNA Sequencing Reveals Initiating Cell Circuits in Pulmonary Inflammation
Source: ACS Nano. 2025 Nov 3;19(45):39139–56. doi: 10.1021/acsnano.5c12054 (PMC12632174; doi:10.1021/acsnano.5c12054)
Supplement: Supplementary file 2 [file nn5c12054_si_002.pdf]

ToxAtlas webtool tutorial

1. Title

Gene expression mapping of carbon-based nanomaterial-specific response patterns in acute lung inflammation

2. Motivation and overview

This tutorial guides you through the use of a **web-based ToxAtlas**, an interactive application for exploring and visualizing gene expression of carbon-based nanomaterial (CBN)-specific response patterns during acute lung inflammation in our single-cell RNA sequencing data. This is a brief overview on how to navigate the interface, generate plots, and interpret results.

3. Getting started

- A browser (Chrome/Firefox/Safari etc.)
- Access the ToxAtlas *via*:

  https://organoidtox.shinyapps.io/nanoparticle_only_exposure_app/

Note: The ToxAtlas is ready to use without registration or login. This webpage is made using ShinyCell.

What you see on the main page:

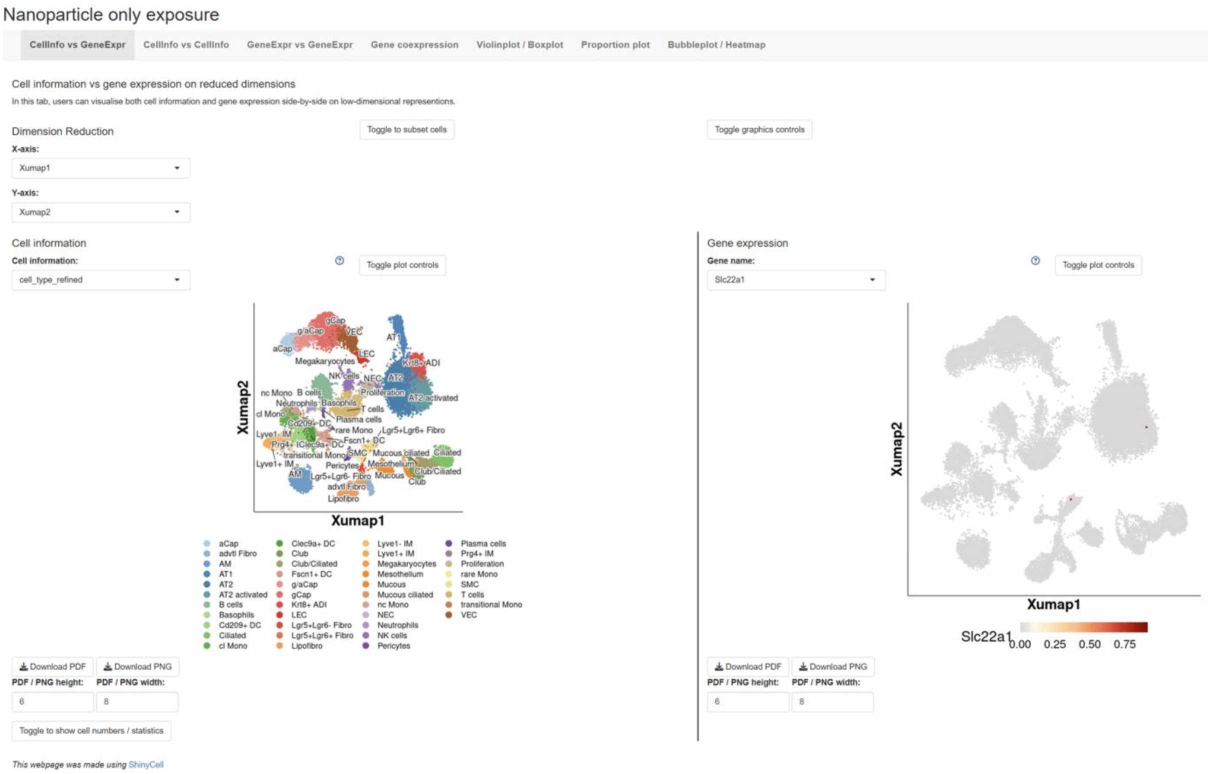

4. Interface Overview

There are seven tabs enabling you to investigate gene expression *via* different comparisons:

- "CellInfor vs GeneExpr": Cell information vs gene expression on reduced dimensions. In this tab, users can visualise both cell information and gene expression side-by-side on low-dimensional representations.
- "CellInfor vs CellInfor": Cell information vs cell information on dimension reduction. In this tab, users can visualise and compare cell information side-by-side on low-dimensional representations.
- "GeneExpr vs GeneExpr": Gene expression vs gene expression on dimension reduction. In this tab, users can visualise gene expression side-by-side on low-dimensional representations.
- "Gene coexpression": Coexpression of two genes on reduced dimensions. In this tab, users can visualise the coexpression of two genes on low-dimensional representations.
- "Violinplot / Boxplot": Cell information / gene expression violin plot / box plot. In this tab, users can visualise the gene expression or continuous cell information (*e.g.* Number of UMIs / module score) across groups of cells (*e.g.* library / clusters).
- "Proportion plot": Proportion / cell numbers across different types of cell information. In this tab, users can visualise the composition of single cells based on one discrete cell information across another discrete cell information. Usage examples include the library or cellcycle composition across clusters.
- "Bubbleplot / Heatmap": Gene expression bubbleplot / heatmap. In this tab, users can visualise the gene expression patterns of multiple genes grouped by categorical cell information (*e.g*. library / cluster). The normalised expressions are averaged, log-transformed and then plotted.

5. Performing Basic Tasks

- Visualizing clusters or cell types
- Exploring gene expression and co-expression
- Exploring treatment effect
- Exploring cell compositions

6. Example Snippet:

Generating a UMAP Plot of Cxcl1 gene expression

(1) Go to the "CellInfor vs GeneExpr" tab.

(2) Keep dimension reduction with "Xumap1" and "Xumap2", respectively.

(3) In "Cell Information", select your desired function: "cell_type_refined", "timepoint", "meta_celltype", "treat_time", "Xumap1" and "Xumap2".

(4) Go to the "Gene expression" option on the right side of the page, type in "Cxcl1" and select the correct gene.

(5) A UMAP is displayed (as below).

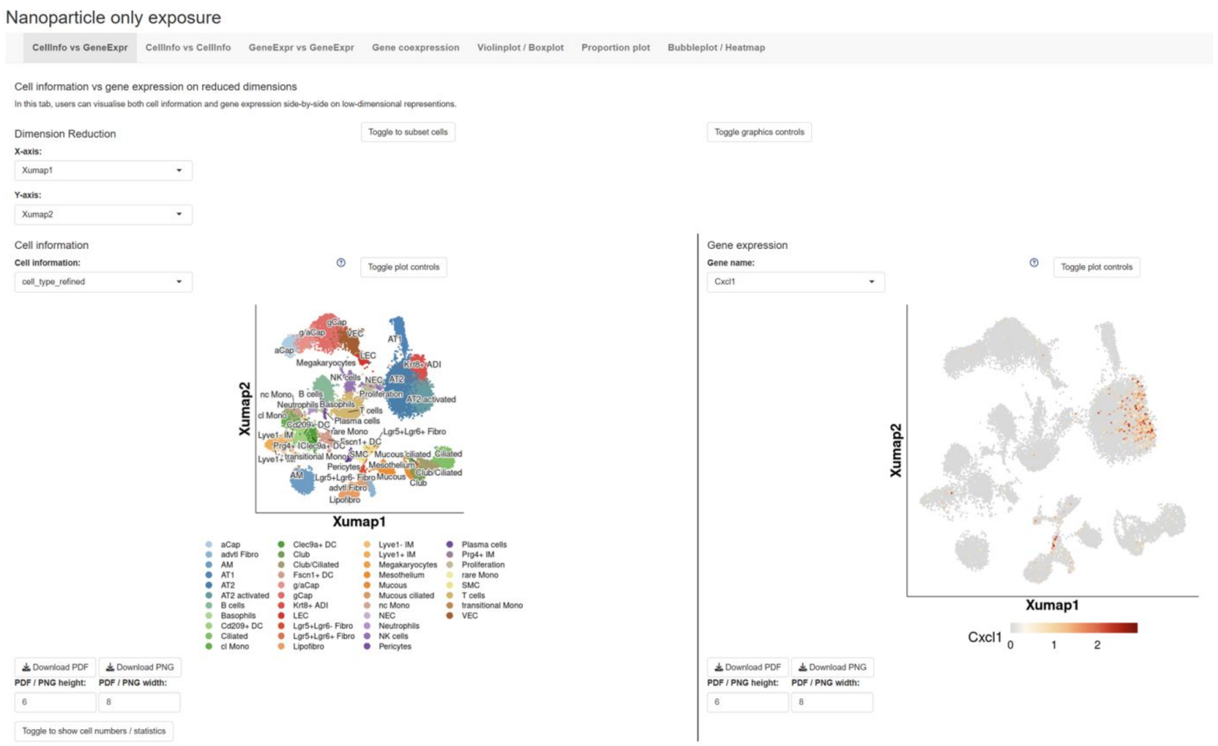

Generating a UMAP Plot of Csf2 and Cxcl1 gene coexpression

(1) Go to the “Gene coexpression” tab.

(2) Keep dimension reduction with “Xumap1” and “Xumap2”, respectively.

(3) Go to the “Gene expression” option on the right side of the page, type in “Csf2” and “Cxcl1” and select the correct gene.

(4) A UMAP is displayed (as below), here the co-expression of *Csf2* (blue) and *Cxcl1* (red) are shown.

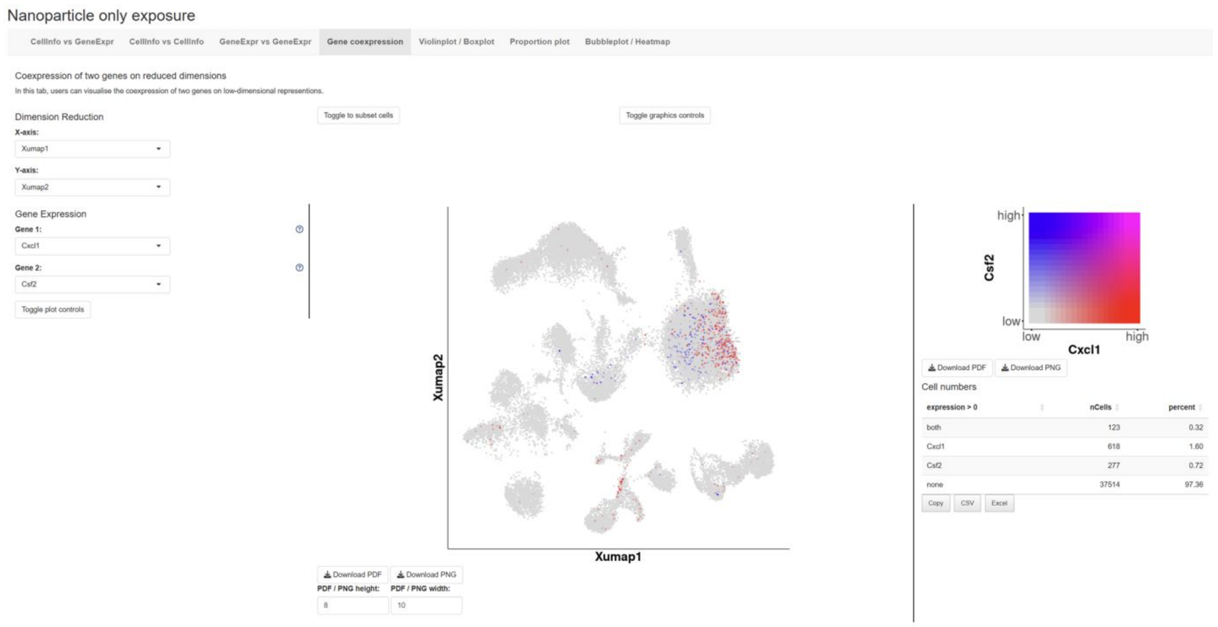

Generate a Bubbleplot to display gene expression between treatments

Here, we show the example of two specific cytokine genes for each CBN (CNP: *Cxcl1*, *Csf2*; DWCNT: *Ccl2*, *Ccl3*; MWCNT: *Ccl11*, *Ccl19*)

(1) Go to the "Bubbleplot/Heatmap" tab;

(2) Find the box under "List of gene names (Max 50 genes, separated by , or ; or newline):" and type in Cxcl1, Csf2, Ccl2, Ccl3, Ccl11, Ccl19;

(3) Select "Group by" as "treat_time" or what categories as you wish to visualize the data;

(4) Select "Plot type" as "Bubbleplot" or "Heatmap" as you wish to visualize the data;

(5) We recommend using "Scale gene expression", you can also decide to select "Cluster rows (genes)" and "Cluster columns (samples)" as you need;

(6) A bubbleplot is displayed (below).

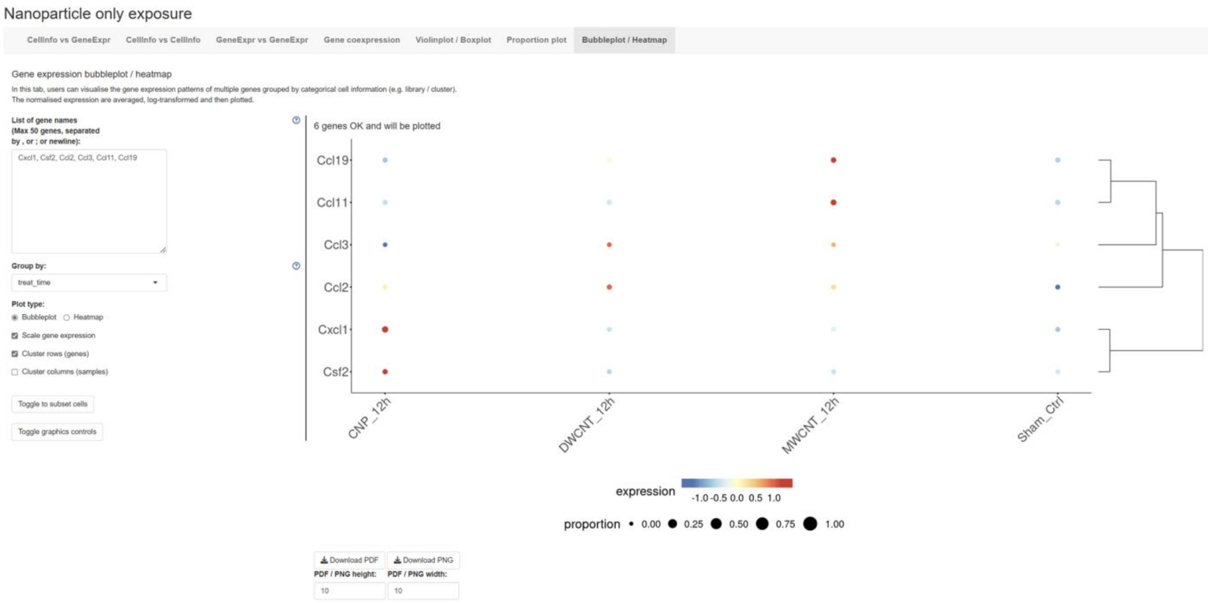

**Suggestions*

(1) Click "Toggle to subset cells", users can set a subset of any cell information included in the tool. As shown below, users can select which cells they want to be displayed on the UMAP.

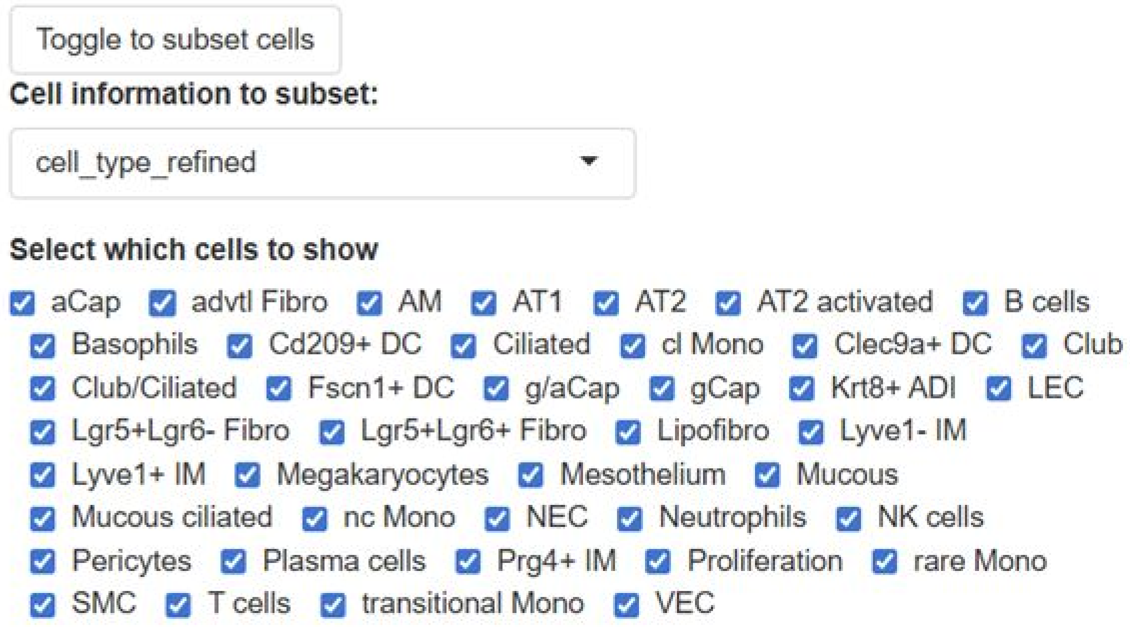

(2) The default colour palette is "White-Red", users are able to change to the other two options by clicking "Toggle plot controls". Besides, showing the highly expressed cells / dots on top of the rest on the UMAP, please set Plot order to "Max-1st" (the default setting).

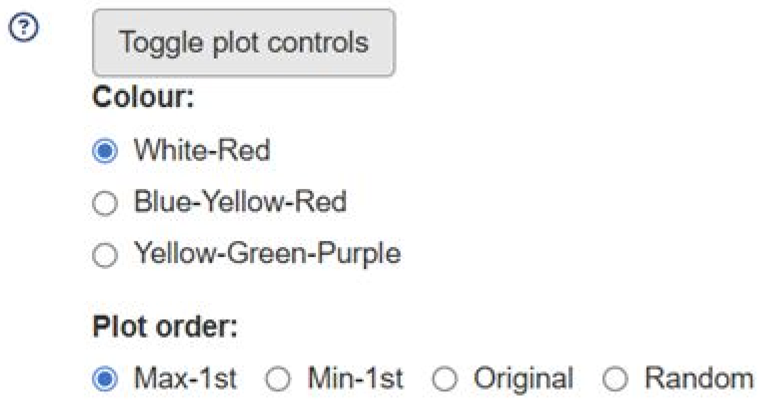

7. Interpreting Results

The UMAP on the left shows the 41 identified cell types ("cell_type_refined") annotated in the project; the UMAP on the right shows the localization of Cxcl to Krt8+ ADI and AT2 activated cells.

8. Saving and Exporting

It is possible to save plots by clicking "Download PDF" or "Download PNG" under the UMAP. Users are able to set the height and width of the exported file.

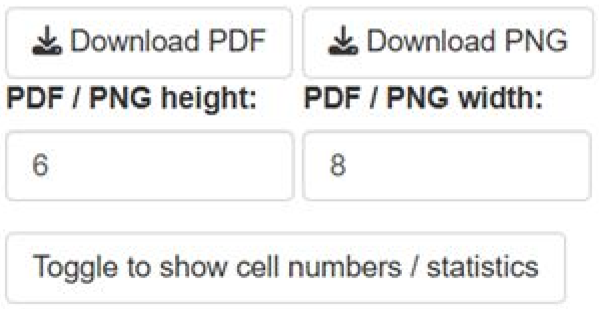

9. Resources

For feedback please contact: Dr. Tobias Stoeger tobias.stoeger@helmholtz-munich.de

Institute of Lung Health and Immunity (LHI) / Comprehensive Pneumology Center (CPC), Helmholtz Munich, Germany

Important: Please cite this publication when using exported plots from our ToxAtlas webtool for publications
